# Aging Reduces Intestinal Stem Cell Activity in Killifish and Intermittent Fasting Reverses Intestinal Gene Expression Patterns

**DOI:** 10.1111/acel.70229

**Published:** 2025-09-22

**Authors:** Michael Kothmayer, Sylvia Laffer, Philipp Widmayer, Elmar E. Ebner, Fehima Ugarak, David Martin, Stefan H. Geyer, Kareem Elsayad, Wolfgang J. Weninger, Sabine Lagger, Klara Weipoltshammer, Oliver Pusch, Christian Schöfer

**Affiliations:** ^1^ Division of Cell and Developmental Biology, Center for Anatomy and Cell Biology Medical University of Vienna Vienna Austria; ^2^ Unit of Laboratory Animal Pathology, Center of Pathobiology University of Veterinary Medicine Vienna Vienna Austria; ^3^ Division of Anatomy, Center for Anatomy and Cell Biology Medical University of Vienna Vienna Austria; ^4^ Institut FEMTO‐ST Université de Franche‐Comté, CNRS Besançon France

**Keywords:** aging, intestinal stem cells, killifish, nutrition, rejuvenation, senescence, stomach

## Abstract

The process of aging is associated with a decline in cell, tissue, and organ function, leading to a range of health problems. Increasing evidence indicates that dietary restriction can counteract age‐dependent effects and improve health and longevity in whole organisms, but less is known about the influence of aging and the impact of nutrition on individual organs of an organism. In this study, we examined the intestine of the very short‐lived aging model system, the African turquoise killifish ( *Nothobranchius furzeri* ), throughout its lifetime. We investigated the effects of age and nutrition on the preservation of gut tissue at stages corresponding to human neonatal, adolescent, adult, and old age, and integrated morphological measurements, histology, and transcriptomics. The intestinal mucosa is characterized by folds and intervening interfold regions, where intestinal stem cells localize. The stem cells occur in clusters, and the cycle time of stem cells increases with age. We also observed a reduction in intestinal length and volume with age. Age‐dependent transcriptomic profiling revealed significant changes in the expression of peripheral circadian clock genes and stem cell niche markers. Notably, the majority of these genes maintained their adult gene expression levels in old age following intermittent fasting during adulthood. Therefore, our results demonstrate that the decline in structural intestinal tissue homeostasis is associated with a decline in stem cell activity that can be counteracted by intermittent fasting. Since the intestinal mucosa of killifish is similar to that of mammals, the results of this study can be translated to general gut biology.

## Graphical abstract

Intestinal stem cells occur in clusters, and the cycle time of stem cells increases with age. The age‐dependent decline in structural intestinal tissue homeostasis is associated with a decline in stem cell activity. Aging causes significant changes in intestinal gene expression; intermittent fasting from adulthood onwards results in a gene expression pattern in old age that resembles that of adult fish.

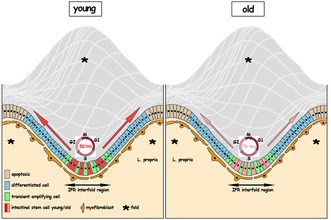

## 1 Introduction

The gastrointestinal tract has unique morphological and functional characteristics to fulfill its role as a supplier and processor of nutrients throughout a lifetime (Gehart and Clevers 2019). The process of absorption is metabolically demanding for the intestinal mucosa, resulting in a high turnover of epithelial cells. A robust regenerative capacity is essential to maintain this dynamic system and thus preserve the tissue integrity of the gut tube. In mammals, regeneration originates from intestinal stem cells (ISCs), which are located in specialized stem cell compartments, the crypts, where they give rise to all differentiated cell types of the epithelium. A substantial body of knowledge exists on the mechanisms that define ISCs, the stem cell niche and the factors involved in differentiation (Cheng and Leblond 1974; for a review see Baulies et al. 2020). Mammalian ISCs are generally regarded as a cell population that maintains itself by asymmetric cell division and is supported by neighboring Paneth cells. At least two populations of ISCs have been identified. The ISCs located at the base of crypts represent a fast‐dividing ISC population and can be identified by the expression of LGR5, whereas those located adjacent to Paneth cells at position +4 of crypts are slow cycling and express HOPX. In contrast, the recruitment of differentiated cells that regain a progenitor‐like cell fate illustrates a high degree of plasticity in tissue regeneration (de Sousa and de Sauvage 2019). Crypts are only present in mammals and birds and are absent in other vertebrate clades. The dynamics of the intestinal epithelium and its homeostasis in exothermic vertebrates remain poorly understood. In fish, the dynamics of the intestinal epithelium have been studied in adult zebrafish and medaka (Aghaallaei et al. 2016; Tavakoli et al. 2022). The ISCs have been identified to occur in stem cell clusters that are dependent on signaling from the stem cell niche in the lamina propria beneath the epithelium.

The continuous renewal of the intestinal epithelium throughout life depends on the proliferative capacity of intestinal stem cells (ISC), which is essential for functional and structural tissue homeostasis (Aghaallaei et al. 2016; Gehart and Clevers 2019). Aging is the result of cell autonomous determinants as well as external drivers such as nutrition. A key factor for the progression of aging is cellular damage (for a review see (Lopez‐Otin et al. 2023)). In humans, the process of aging is associated with a reduction in the absorptive capacity of the gut, which can result in malnutrition in the elderly (Nagaratnam 2019). The regenerative capacity of ISCs during aging has been intensively studied in humans, mice (Gebert et al. 2020; Mihaylova et al. 2018; Pentinmikko et al. 2019; for a review see Gehart and Clevers 2019), medaka (Aghaallaei et al. 2016) and *Drosophila* (Biteau et al. 2010); for a review see (Mank and Rideout 2021). In general, a dysregulation of epithelial homeostasis during aging has been observed. While an increase in proliferation was found in *Drosophila*, a reduction in regenerative capacity was described in mice. In order to facilitate longitudinal studies, it is preferable to use a model system that has a short lifespan but is phylogenetically relatively close to humans, that is, a vertebrate species. The turquoise killifish *Nothobranchius furzeri* ; Jubb 1971 (hereafter *N. furzeri* or killifish) is the vertebrate model system with the shortest lifespan and is a perfect organism for longitudinal aging studies (Platzer and Englert 2016; Terzibasi et al. 2007). Despite the very short maximum lifespan (defined as 10% survival rate) of 20 weeks (strain GRZ), the fish undergoes significant age‐related degenerative processes similar to those observed in mammals, including humans. These processes also include a reduction in food intake in old fish.

Nutrition has been identified as an important factor influencing the process of aging. The quantity of food consumed has a profound effect on the health status of organisms and can influence life expectancy. A reduction in food intake in laboratory animals has been demonstrated to correlate positively with health and longevity, a phenomenon that is conserved across species (Gebert et al. 2020; Fontana and Partridge 2015; Green et al. 2022). There is evidence that dietary restriction attenuates age‐dependent diseases in humans (Waziry et al. 2023) and in killifish (McKay et al. 2022; Valenzano et al. 2006). Intermittent fasting (IF), conducted as fasting every other day, promotes health status and longevity in *N. furzeri* (Terzibasi et al. 2009). Furthermore, dietary interventions have also been shown to affect the gut tube itself. In rodents, dietary restriction has been shown to enhance intestinal stem cell activity (Nalapareddy et al. 2022; Yilmaz et al. 2012) and the potential for ISC differentiation (Gebert et al. 2020).

In particular, IF improves ISC function in mice through the induction of a robust fatty acid oxidation program (Mihaylova et al. 2018). It is therefore important to study the effects of aging and nutrition on the intestine throughout life. Such studies are rare in mammals due to their long lifespans. A longitudinal proteomics study in mice demonstrated that aging is associated with altered expression of factors indicating malabsorption, altered cell proliferation and epithelial cell composition, and that dietary restriction induces a gene expression pattern indicating partial restoration of the epithelium by promoting the capacity for ISC differentiation (Gebert et al. 2020).

This study examines the intestine of the short‐lived vertebrate aging model system, the African turquoise killifish *Nothobranchius furzeri* . The basic anatomy of the killifish gut tube has recently been published (Borgonovo et al. 2024; Dyková et al. 2022). Here, the intestinal tube of male fish was studied throughout their lifetime, using ages corresponding to human neonatal age, adolescence, adulthood, and old age. Morphological and transcriptomics approaches were integrated with nutrition regimes, *ad‐libitum* feeding, and intermittent fasting. We identified ISCs, which are located in clusters, and found that stem cell activity declines with age, as reflected by altered expression of genes involved in epithelial dynamics. Notably, these age‐dependent changes in gene expression were attenuated by intermittent fasting.

## 2 Results

### 2.1 The Gross Anatomy of the Intestinal Tube Is Consistent With That of Agastric Fish

In this study, the gut tube was analyzed across the entire lifespan of male animals at specific age stages corresponding to the human stages newborn (3–4 days old; larvae), adolescence (3–5 weeks), adult (12–13 weeks), and old age (18–20 weeks).

**FIGURE 1:**
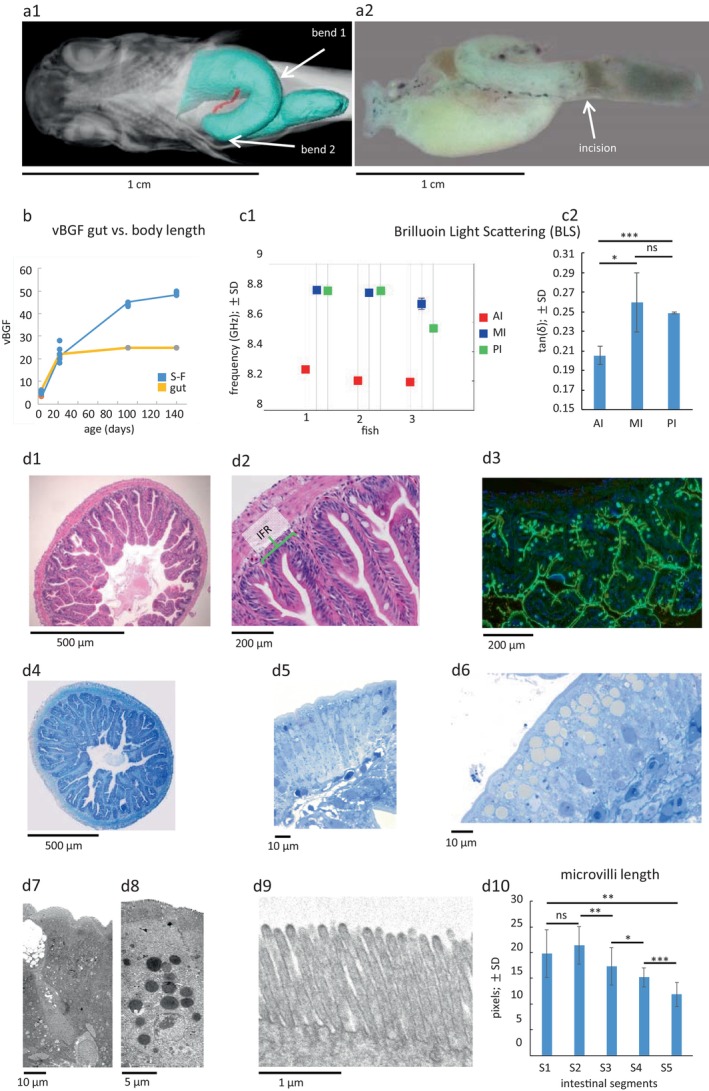
Morphology of the *N. furzeri* gut tube. (a) a1 shows 5 weeks old fish (virtual reconstruction of serial sections; HREM) with the segmented, two‐bended intestinal tube indicated in turquoise (see also Video S1) and the distal ductus choledochus (red) close to its entry into the intestine just before the first bend; ventral view; a2 shows native extracted intestine with incision between MI and the darkish PI (most of the liver has been removed); dorsal view. (b) vBGF, von Bertalanffy growth function showing decoupling of body length (blue; S‐F: Snout‐to‐caudal fin root) and gut tube length (yellow) growth during aging. (c) c1 Brilluoin measurement (loss tangent) of frequencies (GHz) at three different positions of the gut tube (AI (red), MI (blue), PI (green)) in three animals (*n* = 3); c2 graph shows significantly lower tan (*δ*) in the intestinal bulb (AI) indicating higher elasticity there (the lower the frequency the higher the elasticity), while no significant difference was found between the loss tangent values of MI and PI; bars represent mean values, whiskers ± SD. (d) d1, d2: Histology shows folds and interfold regions (IFR); crypts are absent; cross section, HE staining; d3: Goblet cells are abundant in the intestine as shown with WGA staining (green; DAPI: Blue); d4: Semi‐thin cross section; d5: Enterocytes of MI showing abundant smaller granules in the apical portion of cells; semi‐thin section; d6: PI numerous large vacuoles are present in PI enterocytes; semi‐thin section; d7: Ultrastructure of enterocytes and a goblet cell from MI; d8: Ultrastructure of enterocytes from PI (note large vacuoles); d9: Enterocyte with well‐developed brush border; d10: The microvilli size decreases significantly from anterior to posterior; bars represent mean values, whiskers ± SD. *p < 0.05; **p < 0.01; ***p < 0.001; ns, not significant; Student's paired *t*‐test with a two‐tailed distribution.

**FIGURE 2:**
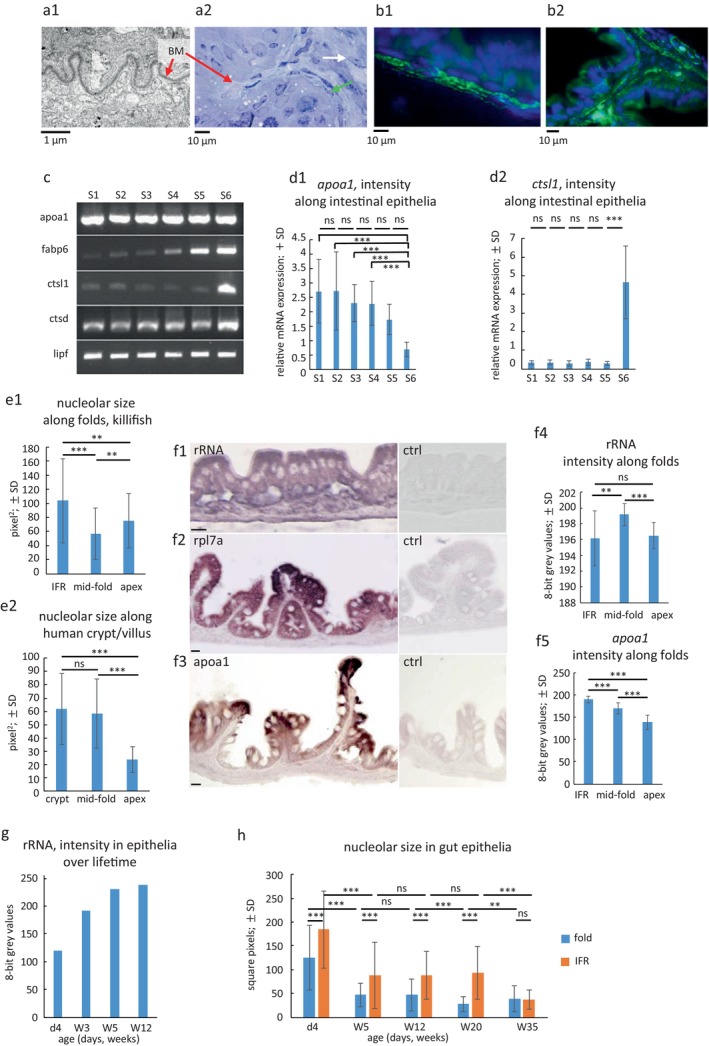
Functional heterogeneity of gene expression along folds, the entire gut tube, and through lifetime. (a) a1: EM micrograph shows prominent basal membrane (BM; red arrow); a2: Semi‐thin image shows adjacent lamina propria with different morphology of those cells (green arrow) that contact the basal membrane (red arrow) versus the reminder fibroblasts (white arrow). (b) b1, b2: Anti‐SMA staining (green) shows label directly below the epithelium (DAPI blue), with no cell layer in between, likely to be myofibroblasts. (c) expression (RT‐PCR) of selected factors involved in nutrient uptake in segments along the gut tube (acc. to scheme Figure S1b1–3); three pooled samples per segment. (d) qPCR of *apoa1* (d1) and *ctsl1* (d2) expression shows decreasing expression of *apoa1* towards caudal parts (significances: Upper layer pairwise comparison, lower layer shows highly significant differences of S1–S4 compared to S6) whereas *ctsl1* is almost exclusively expressed in PI; bars represent mean values, whiskers ± SD; pooled samples from three AL animals, two independent experiments with triplicates each. *p < 0.05; **p < 0.01; ***p < 0.001; ns, not significant; one‐way Kruskal‐Wallis‐ANOVA followed by the Dunn's test (*apoa1*) and ANOVA followed by Tukey's test (*ctsl1*). (e) e1: Nucleolar sizes (area) change from interfold region (IFR) towards the tip of folds in adult killifish; in humans, the nucleolus is smallest at the tip of villi and is larger but of constant size in crypts and along folds (e2); bars represent mean values, whiskers ± SD. (f) f1, 4: Detection of ribosomes in killifish by ISH (rRNA) and f2 by IHC (ribosomal protein l7a; rpl7a) shows differing signal intensities along the epithelium (ctrl: Controls) inversely correlated to the nucleolar size measurement; (8‐bit gray values: 0 = black, 255 = white); f3, 5: Expression of *apoa1* in killifish (ISH) reveals increasing signal density towards the apex of folds. Representative images; bars represent mean values, whiskers ± SD; bars = 20 μm. (g) intensity of rRNA expression (ISH; label intensities on sections) in the whole intestinal epithelium decreases over time. (h) Measurement of nucleolar sizes (area) in the IFR (blue bars) and in the upper parts (yellow bars) of the folds (but not in the apex) confirm the size differences between IFR and folds and show that nucleolar activity is highest in newborn fish but remains constant from adolescence to old age. In centenarian fish (35 weeks) the nucleolar size in the IFR is significantly smaller than in animals of the shorter‐lived animals; bars represent mean values, whiskers ± SD. *p < 0.05; **p < 0.01; ***p < 0.001; ns, not significant; Student's paired *t*‐test with a two‐tailed distribution.

The gut tube of *N. furzeri* is representative of stomachless (agastric) fish. The description of the general anatomy of the gastrointestinal tract follows that proposed in studies of agastric medaka (Aghaallaei et al. 2016) and killifish (Borgonovo et al. 2024). The gut tube shows two bends and consists of an anterior part (AI), which has a wide lumen (also known as intestinal bulb), a middle part (MI) and a distinct, reddish‐brownish colored posterior intestine (PI). The ductus choledochus enters the intestine before the first bend at the end of the AI. After the second bend, the intestinal tube runs straight to the anus (Figure 1a1,2; Video S1). To confirm the hindgut nature of the PI, the fish were incubated in water containing horseradish peroxidase, which is known to be preferentially taken up in the hindgut of fish (Iida and Yamamoto 1984; Noaillac‐Depeyre and Gas 1973; Rombout et al. 1985). Indeed, DAB staining was found to be restricted to the corresponding larger segment (Figure S1a1,2). Furthermore, a preferential expression of cathepsin L (*ctsl1*) was observed in this segment (see below; Figure 2c; d2), which is a marker of colon‐like segments of the intestine in zebrafish and medaka (Aghaallaei et al. 2016; Wang et al. 2010).

This anatomy is invariant between individuals, with the exception of variability observed in the second bend of the intestine. Some fish exhibited a direct upward bend to the dorsal midline, whereas others showed a slight to prominent anterior bend, with the intestinal loop bulging deeply into the liver. As a result, individual variability could be observed in the total length of the intestinal tube. In adult males, approximately one third of the animals studied had a prominent loop oriented towards the cranial side, while two thirds had a more upwardly oriented loop.

### 2.2 Intestinal and Body Growth Become Decoupled at Old Age

The intestine of newly hatched larvae lacked the bends seen in adult animals. The bends developed during the first days after hatching (not shown). Furthermore, histological analysis revealed that the height of the folds increased up to 3 weeks of age, after which no further discernible alterations in the intestinal histology were observed. The length and weight of the animals and of the gut tubes were measured, and the intestinal surface areas of gut tubes were calculated. These values were then compared with the growth of the whole animals during their lifetime. We found lifelong growth in total body length (i.e., measured from the tip of the lower jaw to the root of the caudal fin), although the rate of increase was much reduced in old age (Figure 1b; Figure S1c1–6). The length, diameter, and weight of the gut tube increased significantly up to adulthood (13 weeks). However, a significant decrease in length and diameter was observed between adult and old age. The loss of length seems to be mainly due to a change in the shape of the second bend of the gut tube from a forward‐ to an upward‐directed shape. At 5 weeks of age, the majority of the fish exhibited a forward‐curved gut tube. However, with increasing age, the percentage of fish with this forward bend decreased.

Corresponding to the decrease in gut tube length, a decrease in gut tube weight was found. The surface area of the intestine was then calculated based on the measured amplification due to surface enlargement (Helander and Fandriks 2014) and, consistent with the previous findings, a reduced intestinal surface area was found in old age (Figure S1c2). The observed weight loss of the gut tube was accompanied by a reduced overall weight of old fish (Figure S1c2,5,6; d1,2). Taken together, in our samples length, diameter, volume, and weight of the gut tube increased in parallel with the growth of the whole fish approximately until adulthood (13 weeks). From then on, the intestinal parameters decoupled from body length growth and decreased significantly while the whole body continued to grow, albeit at a slow rate. We hypothesize that the reduced surface area available for nutrient uptake in old age leads to reduced body weight in old age and may have consequences for other age‐related phenomena such as reduced locomotion.

### 2.3 The Killifish Genome Lacks Functional Genes Typical for Stomach

The intestinal tube did not contain complex intra‐ or extra‐epithelial glands in any region. Thus, the AI lacked glands typical of gastric animals and had no gastric sphincters, highlighting the agastric nature of the killifish (Lie et al. 2018; Montesano et al. 2019). Screening of the *N. furzeri* genome in databases (Ensembl release 111; (Harrison et al. 2024)) underlined the agastric nature of the killifish gut tube that is reflected by the absence of stomach‐specific genes known from other gastric species, such as pepsinogens (*pga*, *pgc*), proton pumps (*atp4a*, *atp4b*), mucin‐related factors (*tff1*), or intrinsic factor (*gif*). This is consistent with the agastric zebrafish (Lie et al. 2018) and medaka (own Ensembl search). Putative gastrin (*gast*) and mucin5a (*muc5a*) were found in the killifish and in the medaka genomes, but are absent in zebrafish. Homologs of human gastric lipase f (*lipf*, HGL (human gastric lipase)) were found in all 3 species. To screen for global intestinal gene expression, we performed RNASeq on whole intestinal tubes (Table S1), which confirmed the absence of most of these genes except for *gast*, *muc5a*, and *lipf*. *Lipf* is specifically expressed in the human stomach (Moreau et al. 1989), whereas in zebrafish it is expressed throughout the gut tube (Wang et al. 2010). In this study, we found that the latter is also true for *N. furzeri* (Figure 2c).

### 2.4 The Anterior Intestine Is More Elastic Than the Remaining Gut Tube, Consistent With a Function as Receptacle for Food

The stomach proper is defined by an acidic milieu in the lumen, glands that produce acids and enzymes with optimal activity adapted to the acidity of the lumen, sphincters that restrict the acidic milieu to the stomach, and by the ability of the stomach wall to expand to accumulate ingested food. Although the killifish intestinal bulb lacks all the features of a true stomach, it has a wider lumen, and manual distension with forceps widens the lumen considerably. It has been proposed that the intestinal bulb of agastric fish fulfills the purpose of a food receptacle (Borgonovo et al. 2024). Consequently, the intestinal bulb should be more elastic than the posterior parts of the gut tube. Few reports provide accurate measurements of the mechanical properties of gut tubes in agastric fish (Horton et al. 2022). To test whether there are variations in the extensibility of the killifish gut tube along the longitudinal axis, we used confocal Brillouin Light Scattering (BLS) microspectroscopy. BLS microspectroscopy is a non‐destructive, optical, label‐free technique that can measure the longitudinal elastic and storage moduli of tissues via inelastic light scattering from spontaneous thermal fluctuations (acoustic phonons; (Berne 1977)) and has been increasingly applied to the study of biological systems over the last decade (Elsayad 2021). We performed BLS microspectroscopy measurements in the bulb region (AI), the MI, and the PI of isolated and unfixed gut tubes using the loss tangent method (tan(*δ*); (Elsayad et al. 2020)).

We found that the BLS frequency shift, which is proportional to the longitudinal elastic modulus (Elsayad 2021), is always significantly smaller in the AI than in the MI and PI, suggesting a higher “elasticity” (greater deformability under smaller forces) in the AI than in the caudal intestine (Figure 1c1). The direct relationship between the BLS frequency shift and the elastic modulus is based on the assumption that the refractive index and density do not change significantly (which is a reasonable assumption in this case; (Elsayad 2021)). To ascertain that there is a true change in the mechanical properties, we calculated the mechanical loss tangent (Elsayad et al. 2020), which is independent of these, and a measure of the non‐elastic (viscous) properties relative to the elastic properties. These also showed significantly lower values in AI compared to MI and PI (Figure 1c2), suggesting that while the viscoelastic properties of the stomach region have been preserved, other hallmarks of a true stomach have been lost (see below).

### 2.5 Histology of the Killifish Gastrointestinal Tract

The epithelial lining of the mouth and pharynx contained numerous secretory glands, ranging from single cells (goblet cells) to complex intraepithelial glands and subepithelial glands (Figure S1e1,2). Two pads with pharyngeal teeth were present on the dorsal roof (Figure S2c1,2) (Borgonovo et al. 2024; Tan et al. 2017). The layers of the intestinal wall consisted of a mucosa with a lamina epithelialis composed of high columnar cells (Figure 1d1–8) and a lamina propria, followed by a muscular layer of smooth muscle with an inner circular and an outer longitudinal layer, and a serosa lining the body cavity. The only glands present in the gut tube were goblet cells (Figure 1d3).

The mucosal surface area of the intestine was markedly increased by the formation of folds (Figure 1d1,4; Figure S2a1–3) with intervening interfold regions (IFRs; Figure 1d2). Crypts, higher‐order folds (plicae circulares) and a mucosal musculature (lamina muscularis mucosae) were lacking. The base of the folds is longer than they are wide, so that they appeared paddle‐like. However, the longitudinal extension of these pads can be bent as well as the extension in height, contributing to the irregular appearance of the folds (Figure S2a3). The prominent incision between MI and PI is accompanied by folding of the muscular layer, suggesting a valve function similar to that of the ilio‐caecal valve in mammals (Figure S2b).

Enterocytes displayed a typical brush border at the apical surface. The microvilli were longest in the AI and decreased in length towards the PI (Figure 1d9,10). Goblet cells were abundant but were less numerous than enterocytes (Figure 1d3). The subcellular morphology of enterocytes varied along the rostro‐caudal axis. In the AI and MI, they contained multiple, comparatively small and dark‐stained apical vacuoles, whereas proximal to the MI‐PI junction and in the PI, they showed large bright apical vacuoles (Figure 1d5,6), similar to what has been reported as lysosome‐rich enterocytes in other fishes such as zebrafish (Park et al. 2019). We identified dying cells preferentially at the apex of folds. In the IFR, we found cells of a less differentiated type, and mitotic figures were occasionally seen. Beneath the epithelial layer was a comparatively thick basement membrane (Figure 2a1), followed by the connective tissue of the lamina propria. In the lamina propria, adjacent to the basement membrane, we observed a cell type that is distinct from other fibroblasts, with long processes along the basement membrane. Morphologically, these cells resembled telocytes or myofibroblasts rather than fibroblasts or smooth muscle cells (Figure 2a2). The observed cells stained positively with an antibody against smooth muscle actin (α‐SMA), a marker for smooth muscle cells and myofibroblasts, which is known to be absent from gastrointestinal telocytes (Figure 2b1,2) (Kondo and Kaestner 2019; Vannucchi et al. 2013). Based on their α‐SMA expression, we conclude that these cells are likely to be myofibroblasts.

### 2.6 A Functional Diversity Exists Along the Killifish Gut Tube

Anatomy and histology demonstrated a structural homogeneity of the killifish gut tube, with the exception of the distinction between MI and PI. However, a functional heterogeneity along the longitudinal axis has been reported in zebrafish and medaka gut tubes, which also exhibit a similar homogeneous anatomy (Aghaallaei et al. 2016; Lickwar et al. 2017; Wang et al. 2010). To test whether this is also the case in killifish, we isolated RNA from six segments along the intestine: two segments in the AI, three in the MI, and one in the PI (Figure S1b1–3). Genes known to be either homogeneously distributed or preferentially expressed in certain regions in humans, zebrafish, and medaka were detected by RT‐PCR or qRT‐PCR.

The genes tested included those involved in the uptake and processing of fatty acids and amino acids. These included *apoa1* (apolipoprotein a1), *fabp6* (fatty acid binding protein 6; gastrotropin), *ctsl1* (cathepsin L1), *ctsd* (cathepsin D), and *lipf* (gastric lipase F). *Apoa1* is predominantly expressed in the small intestine of humans and in the anterior intestine of zebrafish and medaka. *Fabp6* is preferentially expressed in the mammalian ileum (Iseki et al. 1993) and upregulated in the posterior part of the gut in medaka (Parmar et al. 2012) and zebrafish (Alves‐Costa et al. 2008; Ma et al. 2022). The distribution of *ctsl1* is comparable to that of *fabp6*, but its expression is much more clearly restricted to the posterior parts in medaka. In contrast, CTSD is expressed in all parts of the human gut tube. Human LIPF expression is restricted to the stomach. However, in zebrafish, *lipf* has been shown to be equally expressed along the entire gut tube (Wang et al. 2010).

RT‐PCR analysis revealed that in killifish *fabp6* and *ctsl1* exhibited high intensity bands in the posterior segments and lower intensities in the anterior parts, whereas *apoa1*, *ctsd*, and *lipf* displayed bands in all segments (Figure 2c). Quantification of *apoa1* expression levels by qRT‐PCR demonstrated a significant decrease in *apoa1* expression towards the posterior end of the gut tube (Figure 2d1). Quantitative RT‐PCR for *ctsl1* in *N. furzeri* confirmed the highly significant increase in expression in the posterior intestine (Figure 2d2). The expression pattern of *ctsl1* in zebrafish led to the conclusion that the posterior intestine can be functionally divided into an ileum‐ and a colon‐like segment, which is similar to the situation observed in mice. Furthermore, high *ctsl1* expression has been associated with bile salt recovery (Lickwar et al. 2017). The significant increase in *ctsl1* expression between segments 5 and 6 in killifish correlates with the distinct morphological boundary between these two segments, indicating an even more pronounced separation of the expression patterns in the hindgut region than observed in zebrafish. Taken together, our results suggest functional heterogeneity in the killifish gut tube, particularly with respect to the distinction between the middle and posterior intestine, which is consistent with studies in zebrafish and medaka (Aghaallaei et al. 2016; Alves‐Costa et al. 2008; Lickwar et al. 2017; Ma et al. 2022; Wang et al. 2010; Wen et al. 2021).

### 2.7 Nucleolar Size of Cycling Intestinal Epithelial Cells Is a Marker of Longevity

Nucleolar size has been shown to be inversely related to lifespan in *C. elegans* and in mammals (Tiku et al. 2017; Tiku and Antebi 2018). In the killifish intestine, we observed a difference in nucleolar size in epithelial cells: in the IFR, nucleoli were significantly larger than along the sides of the folds, where nucleoli were remarkably small. At the apex of the fold, nucleolar size increased again (Figure 2e1). The finding of small nucleoli in cells along the sides of the folds was unexpected and is different from humans (Figure 2e2). To add further to this puzzle, we have recently shown that these cells are senescent (Schöfer et al. 2024), a state that is regarded as non‐proliferative but is metabolically highly active (Blagosklonny 2022). Obviously, a small nucleolus should result in a reduced supply of ribosomes, the main component of the protein synthesis machinery in the cytoplasm of differentiated cells. To test this, we performed RNA in situ hybridization to detect ribosomal RNA (rRNA), which reflected the different nucleolar sizes, showing the strongest signal in the IFR, decreasing along the folds and increasing again at the apex of the folds (Figure 2f1,4). We confirmed these findings by performing immunohistochemistry with an antibody against a ribosomal protein (large ribosomal protein L7a) (Figure 2f2). To understand how this relates to gene expression of factors involved in nutrient uptake, we performed in situ hybridization to detect apolipoprotein a1 (*apoa1*). Measurement along the folds showed that *apoa1* expression was lowest in the IFR and highest at the apex of the folds (Figure 2f3,5). We hypothesize that in the IFR, high production of the protein synthesis machinery is required for cell division and for providing the migrating cells with a sufficient amount of ribosomes to translate moderate levels of factors required for absorption. At the tips of the folds, which are in direct contact with the ingested food, ribosome production is resumed to increase global protein translation levels.

Interestingly, the detection of cellular rRNA content of intestinal epithelia over the lifetime revealed a significant decrease in rRNA amount with age (Figure 2g), suggesting reduced global protein synthesis with age, whereas the relative expression of ribosomal proteins as detected by RNASeq did not change over the lifetime (not shown). A reduction in ribosome occupancy has also been observed in a recent study in the aging killifish brain (Di Fraia et al. 2025). Consistent with this finding, we noticed a significant reduction in nucleolar size at folds in old animals (20 weeks), whereas nucleoli in the IFR remained constant in size, with the exception of newborns, which had significantly larger nucleoli (Figure 2h). In addition, we conducted measurements on a few animals that escaped the 20 week limit of the GRZ strain and lived significantly longer than the rest of the same clutch. We termed animals “centenarians” if they survived beyond the age of 30 weeks. A striking result is the finding of significantly smaller nucleolar sizes in the IFR of centenarians, whereas no significant difference in nucleolar size was noticed at the folds (Figure 2h). As the IFR is the site of cell division, we believe that nucleolar size in the IFR is a proxy for the cell division rate and may reflect an inverse correlation between mitotic activity and lifespan, consistent with (Tiku et al. 2017).

### 2.8 The Interfold Region (IFR) Drives Intestinal Epithelial Regeneration

**FIGURE 3:**
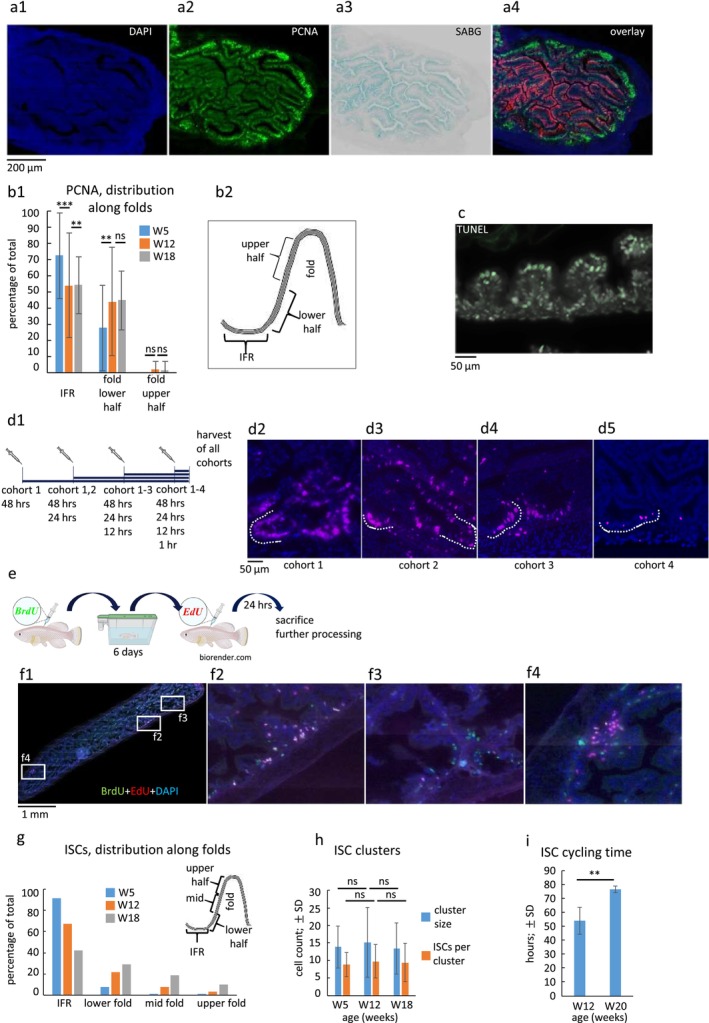
Intestinal stem cells and cellular dynamics in the epithelium of the killifish gut tube. (a) representative image of PCNA staining (green; a2) to depict cycling cells (DAPI blue; a1) shows signal in all IFRs, while folds are devoid of label (AL‐treated animal); a3: SA‐βGal (SABG) staining to detect senescent cells; a4: Overlay (SABG converted to red color) shows PCNA staining in IFR where SABG is absent. (b) during growth (between 5 and 12 weeks), a significant extension of PCNA‐positive cells to the lower half of folds can be detected (b1,2); pairwise Student's *t*‐tests on each topological unit at 3 age stages; bars represent mean values, whiskers ± SD. (c) Apoptosis occurs predominately at the tip of folds (TUNEL green; DAPI white). (d) repetitive injection of EdU in 4 cohorts (scheme in d1) confirmed that cell regeneration starts at IFR (d5, cohort 4) from where cells migrate upwards the folds (d2‐4, cohorts 3–1); dashed lines indicate IFRs in d2‐5. (e) scheme of double‐injection to detect label‐retaining cells, considered to be ISCs. (f) f1: Representative image of small intestine longitudinal section (DAPI blue, BrdU green, EdU red; double‐labeled cells yellow‐white). Three higher magnifications (boxed area in f1) showing an areas with double‐labeled cells (f2), one with cells predominately green (f3; BrdU incorporation) and one area with predominately red cells (f4; EdU incorporation). (g) similar to PCNA distribution (b1), the stem cells extend their occurrence from strictly IFR‐bound at adolescent age (W5; blue bars) towards the folds at old age (W18; gray bars; adult: W12: Yellow bars). (h) cluster sizes (number of all cells in a cluster) and the number of ISCs in the clusters do not change significantly during lifetime; bars represent mean values, whiskers ± SD. (i) Calculation of the cell cycle time of ISCs reveals significant extension of cell cycle time at old age (W20; 76.6 h ±2.3 h) versus adult (W12; 54 h ±9.5 h); bars represent mean values, whiskers ± SD. *p < 0.05; **p < 0.01; ***p < 0.001; ns, not significant; Student's paired *t*‐test with a two‐tailed distribution.

Next, we studied the structural tissue homeostasis in the epithelial lining of the intestine, focusing on the small intestine (AI and MI). Immunostaining of intestinal sections from young animals revealed that the DNA replication marker proliferating‐cell‐nuclear‐antigen (PCNA) was predominantly restricted to the IFRs and became significantly reduced in the epithelium along the folds (Figure 3a1–4; b1,2). During aging, an expansion of PCNA‐positive cells to the folds was observed (Figure 3b1,2). It is noteworthy that all IFRs consistently exhibited comparable labeling intensities (Figure 3a2). To directly demonstrate that proliferation initiates in the IFR, we conducted an injection series with EdU followed by click chemistry (Figure 3d1). Indeed, fish injected with a single dose of EdU 1 day before harvest exhibited labeling restricted to the IFR. In contrast, fish that received multiple injections showed the presence of labeled cells in the epithelium at the folds. Furthermore, an increase in the number of injections resulted in a corresponding increase in the number of labeled cells observed at higher levels in the folds (Figure 3d2–5). These results demonstrate that cell replenishment of the intestinal lining occurs in the IFR. In addition, migration of epithelial cells from the IFR towards the apex of the folds was revealed. This dynamic behavior of the epithelial cells necessitates their elimination by apoptosis, as occurs at the tip of folds or villi in most vertebrates. A TUNEL reaction was applied to directly detect apoptosis, confirming that apoptosis occurs preferentially at the apex of folds (Figure 3c). Taken together, these data demonstrate cell migration from the IFR up the folds and cellular death preferentially at the apex of the folds. Interestingly, we found that the differentiated cells stained positively for the senescence marker SA‐ßGal (senescence associated beta‐galactosidase; SABG) (Figure 3a3), suggesting a possible correlation of senescence with terminal differentiation of epithelial cells. Of note, the onset of senescence is accompanied by a reduction in nucleolar size (shown above), which parallels the decrease in ribosome synthesis.

### 2.9 Intestinal Stem Cells Occur in Clusters in the Interfold Region (IFR)

In mammals, the majority of dividing intestinal epithelial cells belong to the rapidly dividing transient amplifying (TA) cohort. In contrast, the intestinal stem cells (ISCs) divide more slowly and are present in smaller numbers. In an initial attempt to localize ISCs in the killifish intestinal epithelium, we were unable to immunolocalize ISCs using mammalian ISC markers such as *lgr5* (leucine‐rich repeat‐containing G‐protein coupled receptor 5; (Barker et al. 2007)). This is either due to the low antigenicity of commercially available antibodies in killifish or because of the fact that the role of *lgr5* in *N. furzeri* differs from that in mammals (see low gene expression levels in Figure 6c). We therefore attempted to detect label‐retaining cells (LRCs), which are generally considered to be stem cells due to their long turnover period. To achieve this, we applied an injection protocol from *D. rerio* (Glasauer et al. 2021; Li et al. 2020; Tavakoli et al. 2022) using two labeled nucleoside analogues (BrdU and EdU) with an intermediate chase period of 6 days to identify LRCs and obtain information on their cycling behavior (Figure 3e). Paraffin sections of the intestines of such treated animals were subjected to microscopic analysis. EdU‐positive cells indicated cycling cells at the time of euthanasia, whereas careful adjustment of the chase period allowed us to identify LRCs, BrdU‐positive cells. BrdU‐only cells were considered quiescent ISCs, whereas yellow cells, positive for both BrdU and EdU, were considered cycling ISCs. In accordance with previous studies (see above) and for the sake of simplicity, the label‐retaining cells are designated as ISC in this study. However, it should be emphasized that the true nature of these cells, for example, their ability to self‐renew and expand clonally, awaits confirmation. The results demonstrate that (a) ISCs are preferentially localized to the IFR, as expected from PCNA staining, that (b) ISCs are not present in every IFR of the section, although all of them are positively labeled with PCNA, and that (c) ISCs are not homogeneously distributed within IFRs, but rather occur in clusters as inferred from the location of double‐stained, thus proliferating cells (Figure 3f1–4). As we have shown that most of the cells in the IFR are cycling (i.e., they are PCNA‐positive), it is reasonable to assume that the ISCs are located between and intermingled with dividing cells, which most likely correspond to the transient amplifying (TA) cohort known from the mammalian intestine.

### 2.10 The Dynamics of the Epithelial Turnover Decreases at Old Age

The question of whether ISC cluster size and distribution change over the animal's lifetime was addressed by analyzing animals from the following stages of life: 3 weeks (childhood), 5 weeks (adolescence), 10–12 weeks (adult) and 18–20 weeks (old age). The analysis was performed by using the precursor injection approach described above. The evaluation revealed changes in the positioning of the clusters and in the stem cell cycling dynamics. The majority of ISC clusters were consistently detected in the IFR throughout the life of the animals, but we observed a higher frequency of clusters at folds in old fish than in young ones (Figure 3g). Quantification of the cluster sizes revealed that neither the cluster sizes (i.e., the number of cells per cluster that are positive for both BrdU and EdU) nor the number of ISCs per cluster exhibited a significant decrease with age (Figure 3h). We then calculated the cell cycle dynamics of ISCs in adult and old fish using a protocol for mammalian brain tissue based on the double‐labeling approach above using BrdU and EdU (Harris et al. 2018; Martynoga et al. 2005). This protocol relies on the assumption that all evaluated cells of a certain tissue are in a state of active proliferation, which is indeed the case in the IFR of killifish, as evidenced by the PCNA staining. The calculated cycle time for adult (12 weeks) ISCs was approximately 50 h, whereas at the old age (20 weeks) it was about 76 h (Figure 3i). The observed elongation of the cycle time in old‐age ISCs is indicative of a reduced capacity for tissue regeneration, which correlates with the observed reduction in the overall length of the intestine (Figure 1b; Figure S1c1). The shift of ISC localization upwards in the folds (Figure 3g) can be interpreted as a compensatory mechanism to maintain tissue integrity when cell replenishment slows down.

We then asked whether the observed changes in ISC functionality might ultimately lead to impaired intestinal tissue homeostasis at the latest stages of life. To address this, we collected old age post‐mortem fish, that is, those 20 weeks old fish that were alive at the time of daily morning feeding but were found dead later the same day. This strategy enabled us to preserve sufficient intestinal morphology for histological analysis. Indeed, we observed altered epithelial cell morphology in many regions, but never found severe mucosal lesions (Figure S2d1,2), indicating that tissue replenishment was effective until death. This further suggests that reduced intestinal tissue homeostasis is not a life‐limiting factor in killifish.

### 2.11 Dietary Restriction Attenuates Age‐Dependent Changes of Intestinal Gene Expression

**FIGURE 4:**
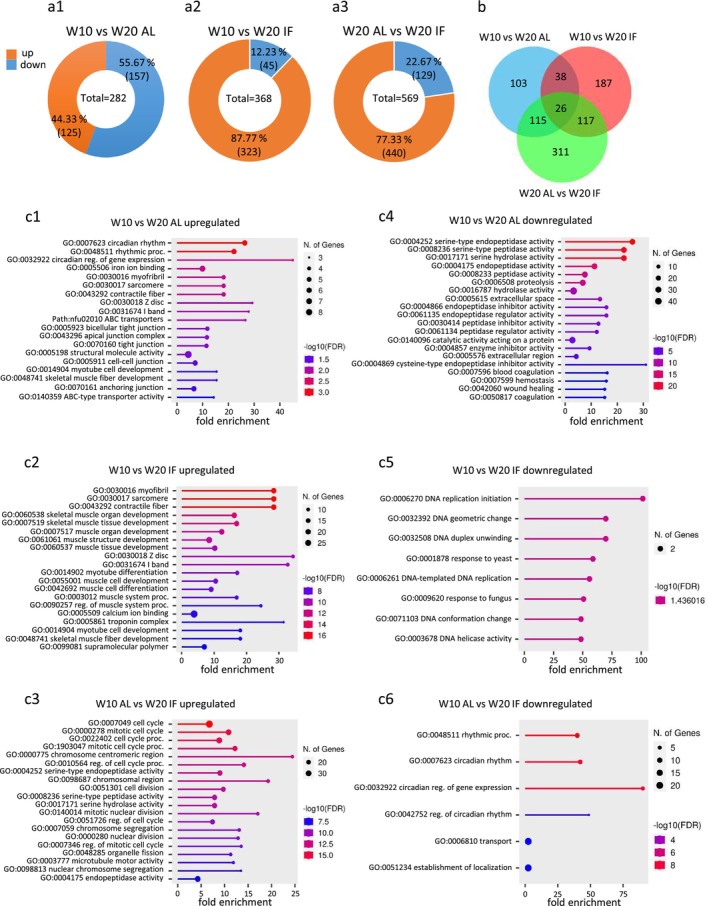
Differentially expressed genes (DEGs) in the killifish intestine depending on age and on diet (RNASeq, DESeq2). (a) total numbers of significantly up‐ (orange) and downregulated (blue) genes, pairwise correlation (a1‐3). The largest DEG‐set is found comparing the W20 AL (*ad libitum*) and W20 IF (intermittent fasting) groups (a3). Log2fold change > 1, >− 1; *p*value adj. < 0.05. (b) combinatorial Venn diagram representing overlapping differentially expressed genes between groups. Biggest effect is observed in old age (W20) comparing AL and IF. Log2fold change > 1, >− 1; *p*value adj. < 0.05. (c) c1‐c6: Gene set enrichment analysis on significant DEGs for each comparison using ShinyGO 0.80 with *N. furzeri* ENSEMBL IDs; for GO‐terms “all available gene sets” was selected.

In order to obtain an unbiased picture of intestinal gene expression during the aging process, we performed a screen for differentially expressed genes (DEGs) using RNASeq on whole intestines from adult (10 weeks) and old (20 weeks) male killifish (Figure 4a1–3; b; Figure S3a1–3; b1‐3). One cohort received *ad libitum* (AL) feeding in a manner consistent with the other analyses in this study, that is, animals were fed twice daily with an excess food supply. A second cohort was subjected to late‐onset dietary restriction by intermittent fasting (IF), with animals receiving excess food only every other day from 10 weeks (adult) to 20 weeks (old). Pairwise comparison of gene expression revealed a prominent impact of IF. 282 significantly differentially expressed genes (adjusted *p*‐value < 0.05; log2fold change > 1, >− 1) were found to be age‐related (10 weeks vs. 20 weeks AL) and 368 were detected under aging and dietary restriction (10 weeks vs. 20 weeks IF, 368 genes). Importantly, the comparison of old animals (20 weeks IF vs. 20 weeks AL) showed approximately twice as many significantly DEGs than age‐related ones (569 genes). Similarly, under IF conditions, the percentage of upregulated genes was much higher than that of downregulated genes (Figure 4a1–3). Comparing DEGs in all three pairs (Venn diagramm Figure 4b) also showed a higher number of DEGs in the two IF comparisons 26 genes were found to be commonly regulated in all three pairs, the majority of which were involved in epithelial homeostasis and barrier function (data not shown). With respect to aging (10 weeks vs. 20 weeks AL), gene ontology (GO) analysis revealed a significant upregulation in gene expression of circadian clock genes and significant downregulation of genes involved in nutrient absorption (Figure 4c1,4). Comparison of 20 weeks AL versus IF showed upregulation of cell cycle regulators and downregulation of circadian genes (Figure 4c3,6). A comparison of 10 weeks versus 20 weeks IF resulted in fewer significant functional differences than the other pairwise comparisons (Figure 4c2,5; see also Figure S2g1–3), indicating that intermittent fasting of older animals approached the gene expression patterns of younger animals. It is also interesting to note that circadian genes *per1* and *per3* show up in the top 20 DEGs in the pairs 10 weeks versus 20 weeks AL and 20 weeks AL versus 20 weeks IF but not in 10 weeks versus 20 weeks IF (Figure S3a1–3; b1‐3).

### 2.12 The Age‐Dependent Change of Intestinal Circadian Gene Expression Is Ameliorated in the IF Cohort

**FIGURE 5:**
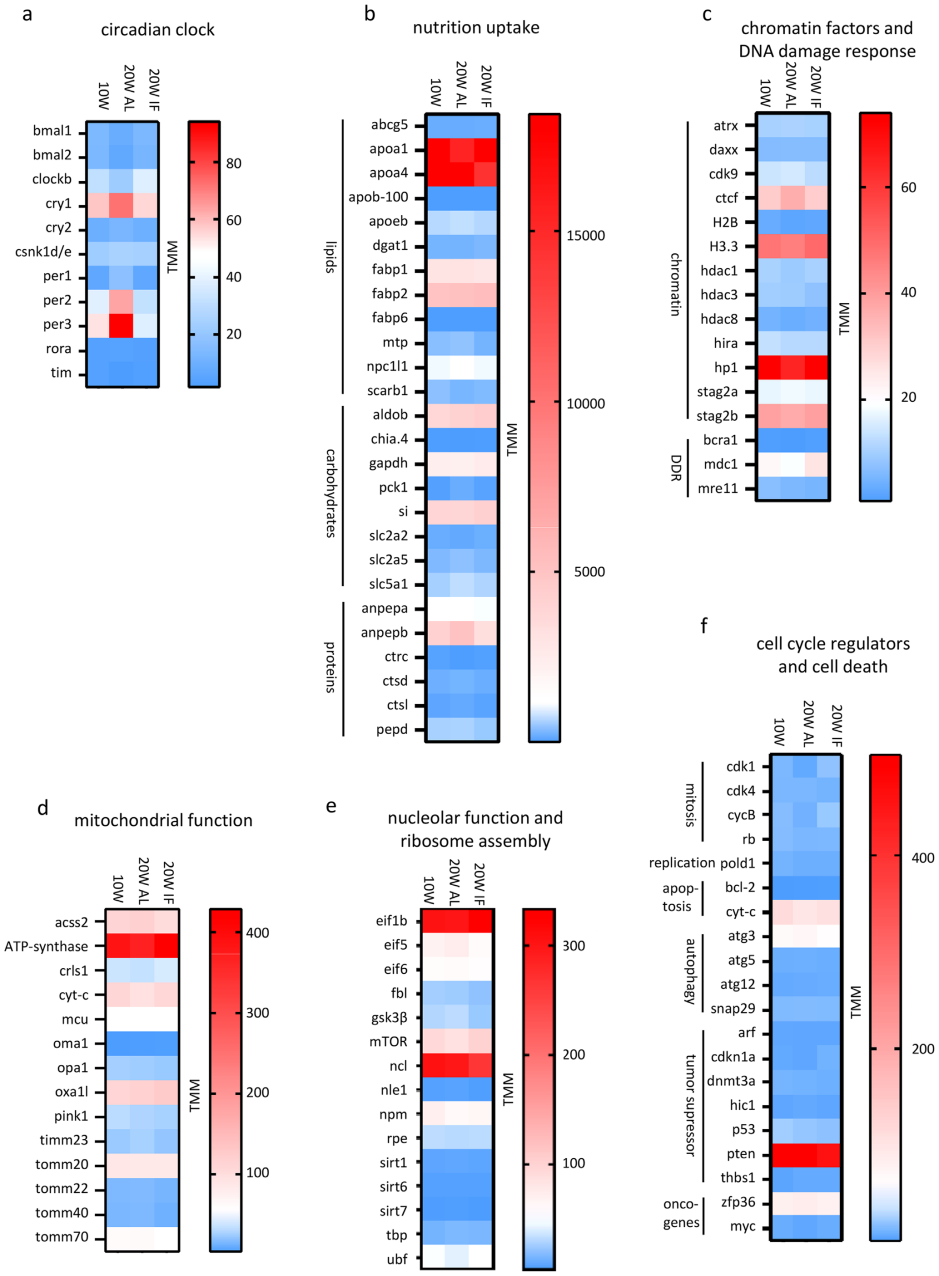
Gene expression differences of manually selected sets of factors known to be part of cellular structures or biological processes in other organisms; for corresponding significant changes in gene expression changes see Figure S4a–f. Raw reads were normalized by TMM (trimmed mean of *M*‐values). (a) all peripheral circadian clock genes show adult gene expression levels in old animals that underwent IF. (b) genes involved in nutrient absorption show many DEGs in the AL group that display adult values in the IF cohort, including *apoa1*, *apoeb*, *mtp*, *npc1l1*, *scarb1*, *pck1*, *slc2a5*, *slc5a1*, *anpepb*, *ctrc*, *ctsd*, *ctsl*. (c) chromatin and DNA damage response (DDR) factors demonstrate adult‐like expression in old IF‐animal in c*dk9*, *ctcf*, *H3.3*, *hdac1*, *hadc3*, *hp1*, *stag2a*, *stag2b*, *mdc1*. (d) factors for mitochondrial functions show adult levels in the old IF cohort in *acss2*, *ATP‐synthase*, *crls1*, *cyt‐c*, *timm23, tomm40*. (e) factors involved in nucleolar functions and ribosome assembly show adult‐like expression levels of *eif5*, *gsk3β*, *mTOR*, *npm*, *sirt1*, and *ubf* in the old IF fish group vs. the AL group. (f) changes in expression of cell cycle and cell death regulators in the old IF animals that resemble that of adult animals include *cdk1*, *cycB*, *cyt‐c*, *atg3*, *cdkn1a*, *hic1*, *zfp36*, and *myc*. Color codes assigned to absolute TMMs.

Age‐dependent and tissue‐specific deregulation of rhythmically expressed circadian genes has been shown in mice (Wolff et al. 2023), human, zebrafish and also in killifish for brain, liver and skin (Barth et al. 2021), however excluding the intestine. It has been concluded that increased variability of circadian gene expression is a common feature of aged tissue and both up‐ and downregulation of circadian genes were observed in different organs. Interestingly, the rhythmic circadian secretion of *wnt3* in Paneth cells of mice organoids is required for the maintenance of ISCs (Matsu‐Ura et al. 2016). A recent study on circadian gene expression in intestines of *Drosophila* revealed a negative correlation between circadian clock function and cell differentiation (Parasram et al. 2024). The peripheral clocks are driven by cell‐autonomous negative feedback loops, which consist of the cyclic expression of genes including *clockb*, *bmal1*, *bmal2*, *cry1*, *cry2*, *csnk1d*, *per 1*, *per 2*, *per 3*, *rora*, and *tim* (Acosta‐Rodriguez et al. 2021). The pairwise comparison of changes in gene expression (e.g., 10 weeks vs. 20 weeks AL vs. 10 weeks vs. 20 weeks IF) of normalized counts after DESeq2 analysis of RNASeq data of the *N. furzeri* intestine revealed significant differences for all clock genes but *rora*. Ten significant DEGs were found for the aging group (W10 versus W20 AL) and seven genes were found significantly deregulated comparing the two old age cohorts (Figure S4a). Importantly, 10 of the 11 selected genes showed no significant differential expression between adult and old age under IF diet (Figure S4a, middle column). Expression levels were highest for *cry1*, *per2*, and *per3* (Figure 5a).

### 2.13 The Age‐Dependent Alteration of Factors for Nutrient Uptake and Cellular Functions Is Mitigated in the IF Cohort

A set of 26 selected factors was assembled that are involved in the absorption and processing of fatty acids, sugars, and amino acids (Figure 5b). RNASeq analysis of these genes revealed age‐ and nutrition‐dependent differential gene expression changes. Twelve 19 out of 26 genes showed no significant differential expression between adult and old age animals under the IF regimen (Figure S4b, middle column). *Apoa1b* and *apo4a* showed by far the highest expression levels (Figure 5b).

Exposure of the intestinal epithelium to the bolus of food and the subsequent processing of nutrients causes considerable stress on the epithelial cells. A set of genes known to be involved in DNA double‐strand repair (DDR) and chromatin regulation was therefore assembled (Figure 5c). Genes that show adult expression levels in old IF animals included c*dk9*, *ctcf*, *H3*.*3*, *hdac1*, *hadc3*, *hp1*, *stag2a*, *stag2b*, and *mdc1* (Figure 5c). Five significant DEGs were found for the aging group (W10 versus W20 AL) and four genes were found significantly deregulated comparing the two old age cohorts (Figure S4c). 15 out of 16 genes showed no significant differential expression between adult and old age animals under the IF regimen (Figure S4c, middle column). Of particular note are the age and nutrition‐dependent changes in the expression of chromatin organizers *ctcf*, *stag2a*, *stag2b*, and the repressive chromatin markers *hp1*, *hadac1*, *hadac3*. An upregulation of the histone variant H3.3 can be associated with transcriptional activity (Ahmad and Henikoff 2002; Snyers et al. 2014). In *ctcf* and *hp1*, the gene expression in old IF animals was maintained at adult expression levels (Figure S4c). The strongest expression was seen in *hp1* (Figure 5c).

Some of the 14 selected mitochondrial factors, *acss2*, *ATP‐synthase*, *crls1*, *cyt‐c*, and *timm23* showed adult gene expression levels in old IF animals (Figure 5d). Only two significant DEGs were found for the aging group (W10 versus W20 AL) and three genes were found significantly deregulated comparing the two old age cohorts (Figure S4d). Eleven out of 14 genes showed no significant changes in expression between adult and old age animals under IF treatment (Figure S4d, middle column); *ATP‐synthase* showed the highest expression level (Figure 5d).

We had observed prominent alterations in nucleolar size and in cytoplasmic ribosome occupancy (see above). In order to see whether these alterations were reflected in changes in factors for ribosomal gene expression and factors required for protein translation, a set of 15 genes was compiled. Genes that show adult expression levels in old IF animals included *eif5*, *gsk3β*, *mTOR*, *npm*, *sirt1*, and *ubf* (Figure 5e). Five significant DEGs were found for the aging group (W10 versus W20 AL) and six genes were found significantly deregulated comparing the two old age cohorts (Figure S4e). 12 out of 15 genes showed no significant differential expression changes between adult and old age animals under the IF treatment (Figure S4e, middle column). *Ubf* is directly involved in rRNA transcription, whereas *mTOR*, a master regulator of nucleolar activity linking more specifically nutrient sensing to ribosome production, is upregulated in the gene set comparing the two old age cohorts. The highest expression levels were found for *ncl* and *eif1b* (Figure 5e).

The processes of aging and nucleolar activity are linked to cell turnover. Therefore, a set of 20 genes with markers of cell cycle regulation, autophagy, and cell death was assembled. Gene expression levels in the old IF animals that resemble those of adult animals include *cdk1*, *cycB*, *cyt‐c*, *atg3*, *cdkn1a*, *hic1*, *zfp36*, and *myc* (Figure 5f). Five significant DEGs were found for the aging group (W10 versus W20 AL) and four genes were found significantly deregulated comparing the two old age cohorts (Figure S4f). Nineteen out of 20 genes showed no significant differential expression changes between adult and old age animals under IF regimen (Figure S4f, middle column). Interestingly, adult expression in the IF group was found in cell cycle promoting factors, for example, *cdk1*, *cycB*, and *myc*, but also in cell cycle inhibitors such as *cdkn1a* (*p21*). The change in gene expression of the interacting cell cycle regulators *cdk1* and *cycB* differs significantly depending on the diet, whereas this is not the case in the interacting genes *cdk4* and *rb*. *Pten* shows the highest expression level (Figure 5f).

**FIGURE 6:**
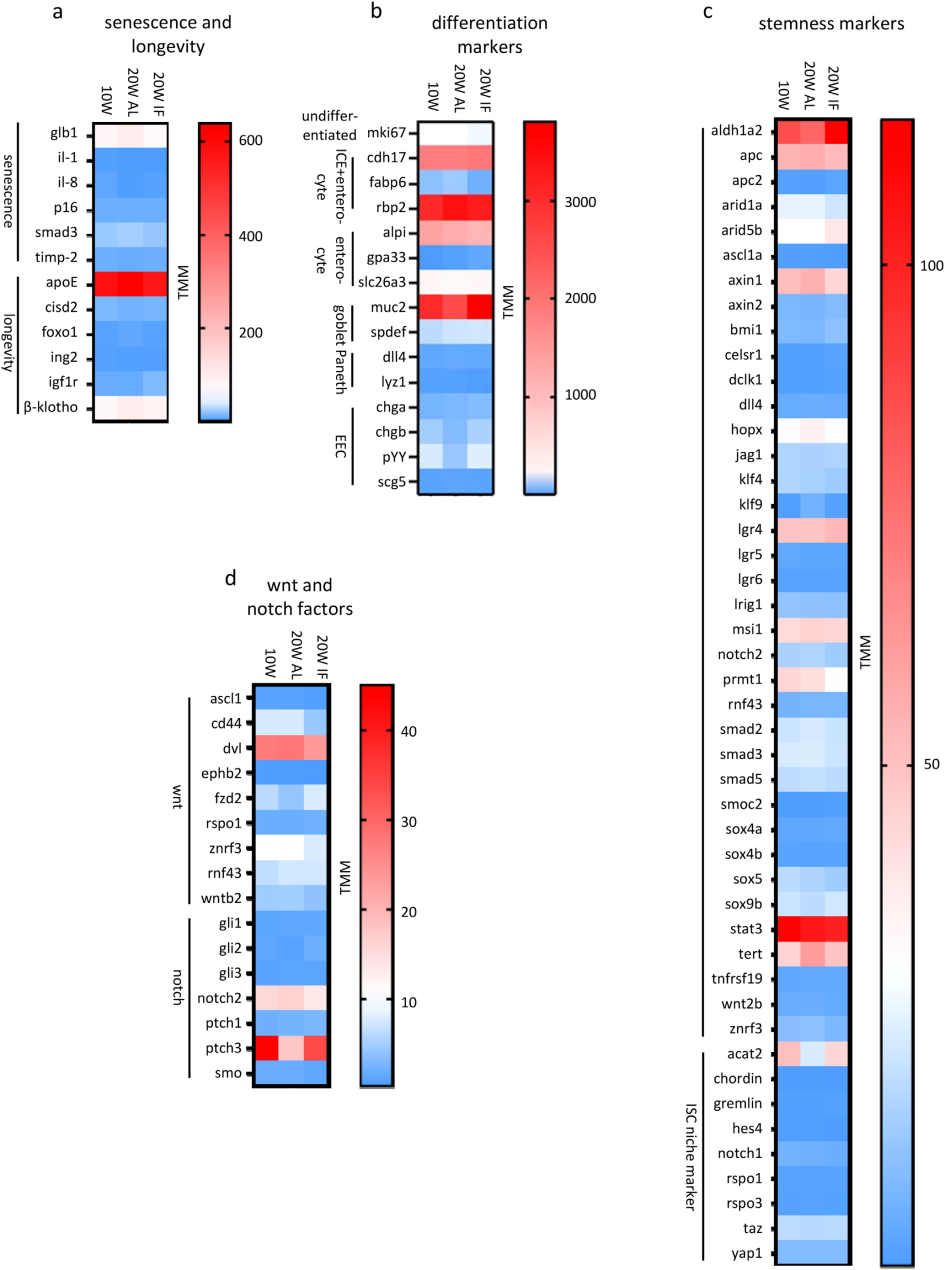
Gene expression differences of manually selected sets of factors known to be part of cellular structures or biological processes in other organisms; for corresponding significant changes in gene expression changes see Figure S5a–d. Raw reads were normalized by TMM (trimmed mean of *M*‐values). (a) the expression levels of senescence and longevity markers *glb1*, *il‐8* and *foxo1* are retained in the old fish of the IF group versus the AL group. (b) changes in expression of differentiation markers *fabp6, rbp2, slc26a3*, *muc2*, *dll4* and, particularly, *chgb* and *pYY* show adult levels in old fish of the IF group in contrast to the AL group. (c) ISC stemness genes presenting adult expression in old age animals of the IF‐group as opposed to the AL animals include *aldh1a2*, *dll4*, *hopx*, *klf9*, *notch2*, *sox9b*, *tert*, and *acat2*. (d) the expression levels of adult Wnt and Notch pathway factors *dvl*, *fzd2*, *znrf3*, *wntb2*, *notch2,* and *ptch3* are retained in the old fish of the IF group. Color codes assigned to absolute TMMs.

Aging is also linked to cellular senescence and longevity. A set of 12 marker genes was established, of which amongst others *smad3, cisd2, glb1*, *il‐8, chga*, and *foxo1* exhibited adult expression levels in old fish under the IF regimen (Figure 6a). Five significant DEGs were found for the aging group (W10 versus W20 AL) and five genes were found significantly deregulated comparing the two old age cohorts (Figure S5a). Eleven out of 12 genes showed no significant changes in expression between adult and old animals under the IF regimen (Figure S5a, middle column). *ApoE* shows the highest level of expression (Figure 6a).

### 2.14 The Age‐Dependent Changes in Expression of Factors Involved in Cell Differentiation and Intestinal Epithelial Stemness Approximate Adult Levels in the IF Cohort of Old Fish

Subsequently, the expression of 15 genes commonly used as markers for epithelial cell populations, including undifferentiated cells (transient amplifying cells TA; Ki‐67, *mki67*), enterocytes (*cdh17*, *fabp6*, *rbp2*, *alpi*, *gpa33*, *slc26a3*), goblet cells (*muc2*, *spdef*), and enteroendocrine cells (*chga*, *chgb*, *pYY*, *scg5*) was evaluated. Gene expression levels in the old IF animals that resemble those of adult animals include *fabp6, rbp2, slc26a3*, *muc2, dll4*, *chga*, and, particularly, *chgb* and *pYY* (Figure 6b). Eight significant DEGs were found for the aging group (W10 versus W20 AL) and six genes were found significantly deregulated comparing the two old age cohorts (Figure S5b). Twelve out of 15 genes showed no significant change in expression between adult and old age animals under IF treatment (Figure S5b, middle column). The highest expression levels were found in *muc2*, *rbp2*, *cdh17*, and *alpi* (Figure 6b).

Furthermore, a set of 46 genes known to be markers of ISCs and stem cell niche factors was assembled. Gene expression levels in the old IF animals that resemble those of adult animals include *aldh1a2*, *apc*, *axin1*, *axin2, bmi1, dll4*, *hopx*, *jag1*, *klf9*, *msi1*, *notch2*, *smad2*, *smad5*, *sox9b*, *tert*, *wnt2b, znrf3*, and *acat2* (Figure 6c). Ten significant DEGs were found for the aging group (W10 versus W20 AL) and 13 genes were found significantly deregulated comparing the two old age cohorts (Figure S5c). Thirty‐nine out of 46 genes showed no significant change in expression between adult and old‐age animals under the IF regimen (Figure S5c, middle column). The highest expression levels were found in *stat3*, *aldh1a2*, *apc*, *axin1*, *lgr4*, *tert*, *msi1*, *prmt1*, and *acat2* (Figure 6c).

The differentially expressed genes contain many factors with well‐established functions in ISCs in mammals (for Wnt and Notch pathway factors see below). *Tert* and *hopx* are markers of the slow cycling, +4 population of mammalian ISCs. The niche marker *acat2* has been shown to be a key metabolite regulating ISC self‐renewal and differentiation in mammals and links fatty acid metabolism to ISC function (Wang et al. 2021). It is also noteworthy that *lgr5*, a prominent ISC marker in mammals, showed low expression levels and was not differentially expressed (Figure 6c; Figure S5c).

The Wnt and Notch pathways play important roles in the maintenance of ISCs and in the balance between ISC homeostasis and differentiation in mammals. Combining major genes involved in the Wnt and Notch pathways (including above mentioned ones) revealed five genes, *dvl*, *fzd2*, *znrf3*, *wntb2*, *notch2*, and *ptch3* that showed adult expression levels in old IF animals (Figure 6d). Two significant DEGs were found for the aging group (W10 versus W20 AL) and six genes were found significantly deregulated comparing the two old age cohorts (Figure S5d). Wnt signaling has generally been shown to promote ISC proliferation. With respect to mucosal regeneration, the differential, diet‐dependent expression of *wnt2b* is particularly noteworthy. This is also true for other known promoters of ISC proliferation, the Wnt‐ligand *sox9b* (see Figure 6c), a marker of ISCs in mammals, in zebrafish and in medaka, wnt‐receptor *fzd2*, the positive Wnt regulator *dvl*, and the Wnt‐counteracting *znrf3*. A further interesting finding is the differential expression of the Wnt inhibitor *apc* (see Figure 6c), which may be related to the observed stem cell exhaustion. Similarly, the Notch pathway is another key player in the regulation of ISC proliferation and differentiation. Therefore, the diet‐dependent differential expression of *notch2* and its ligand *dll4* (see Figure 6c) may be beneficial for the maintenance of mucosal homeostasis. 13 out of 16 genes showed no significant change in expression between adult and old age animals under IF regimen (Figure S5d, middle column). The highest expression levels were found in *ptch3*, *dvl*, and *notch2* (Figure 6d).

To obtain precise data on the age‐dependent expression along the lifespan, we performed qRT‐PCR of a selection of genes in newborn, adolescent, adult, and old fish under the AL regimen (Figure S2f1–5). The data obtained were consistent with the RNASeq DEG analysis showing a significant decrease in gene expression between adult and old age for the stem cell niche factor *acat2*, while no significant decrease was detected between adult and old age for *ascl1a*, *rspo1*, *rspo3*, and *sox9b*.

Overall, RNASeq analysis revealed a significant effect of the dietary restriction regime on gene expression in old killifish. Our data indicate that under IF treatment, the expression of many genes in old fish maintains gene expression levels comparable to those observed in adult animals. More than 85% of the genes in the age adult versus old pair on IF diet showed no significant change in gene expression in contrast to 60% when comparing adult and AL fed animals (Figure S2g1–3).

## 3 Discussion

We studied the structural homeostasis of the intestine by morphological and transcriptomic approaches and integrated the effects of two different dietary regimens, *ad‐libitum* (AL) and late‐onset intermittent fasting (IF). We performed the study throughout the lifetime of male animals at stages corresponding to human neonatal, adolescence, adult, and old age.

### 3.1 Gross Anatomy Along Lifetime

The general gut morphology of *N. furzeri* has recently been described (Borgonovo et al. 2024; Dyková et al. 2022) and is typical of agastric fish species, such as medaka and zebrafish. Based on histology, no other cell type than enterocytes and goblet cells could be clearly identified in the mucosal lining of killifish. Other cell types, such as Paneth cells and rodlet cells, a teleost‐specific cell type often observed in the gastrointestinal tract, for example, in zebrafish (Siderits and Bielek 2009), are lacking. The presence of enteroendocrine cells in killifish has been demonstrated by immunostaining (Borgonovo et al. 2024). In zebrafish, single‐cell RNASeq analysis yielded other epithelial cell types known from mammals, such as tuft cells, microfold, and BEST4 cells (Wen et al. 2021; Willms et al. 2022). Similar studies in killifish will certainly expand our knowledge of the different cell types present in the killifish intestinal epithelium. The maintenance of an extensible intestinal bulb wall appears to be relevant as an effective strategy for food consumption when the availability of prey varies over time. The loss of the stomach has occurred several times during the evolution of fishes (Castro et al. 2014; Mhalhel et al. 2023; Wallace et al. 2005) and is accompanied by the loss of functional genes encoding pepsinogens and proton pumps (Castro et al. 2014; Lie et al. 2018), characteristics that we also found in the *N. furzeri* genome. A true stomach is defined by the presence of gastric glands, an acidic environment, the presence of muscular sphincters at both ends, and an extensible stomach wall (Castro et al. 2014; Koelz 1992). With the exception of the extensible wall, none of these features are present in killifish. The reasons for the loss of the stomach remain speculative and may be related to specific diets (Castro et al. 2014). Whether this is the case in *N. furzeri* remains to be elucidated. It is clear that the absence of an apparently non‐essential organ saves a considerable amount of energy. The ductus choledochus enters the small intestine just before the first bend, suggesting that the intestine anterior to this position may have primarily a receptacle function. This view is supported by the observation that this segment is larger in diameter and more “elastic” (i.e., has a lower elastic modulus) than the posterior parts of the intestine, as we demonstrated using Brillouin microspectroscopy.

Interestingly, a decoupling of the growth in length and weight of whole fish and gut tubes was observed from adult stage to old age, which is reflected in a reduction in the total intestinal surface available for nutrient uptake. This reduction in volume is dependent on a reduction in length, which is reflected by shape changes in the second bend, mainly at the expense of MI, while AI and PI remain relatively constant, and by an overall reduction in gut tube diameter. It is likely that the reduction in the intestinal volume plays an important role in many age‐related alterations in killifish, such as for example, sarcopenia. In this study, a clear reduction in ISC functionality was observed with age. We propose that this is the underlying cause of the reduction in gut tube dimensions, which is likely to affect the whole organism. Our previous assumption that the reduced intestinal volume might be the ultimate limiting factor in killifish longevity was contradicted by our morphological analysis of post‐mortem fish, which showed no significant mucosal lesions.

### 3.2 Cellular Senescence Regulates the Dynamics of the Structural Homeostasis of Intestinal Epithelia

An unexpected finding was the low ribosome content of differentiated epithelial cells while they are performing metabolically demanding tasks. Our current explanation for this apparent contradiction is that after differentiation, cells at the base of folds are provided with sufficient amounts of the protein synthesis machinery for their short lives as differentiated cells. However, at the tip of the folds, where the contact between the enterocytes and the ingested food requires increased production of factors for nutrient uptake, ribosome synthesis is resumed, as evidenced by the presence of a larger nucleolus and the highest expression of factors required for nutrient uptake, as exemplified by the detection of *apoa1*.

We have shown that the differentiated cells of the epithelium are in a state of cellular senescence, with the transition from cycling to senescent cells occurring immediately upon exit from the proliferation zone (Schöfer et al. 2024; this study). Cellular senescence is typically associated with the exit of cells from the cell cycle and is thought to be a mechanism to prevent the propagation of cellular damage, such as DNA mutations. Senescent cells have been shown to block pro‐apoptotic pathways and thus accumulate throughout life. Senescence also occurs in differentiated cells, for which a tissue protective function has been proposed (Schöfer et al. 2024). Consistent with this notion, it has been shown that killifish have reduced DNA repair capacity as a consequence of relaxed purifying evolution to adapt to a short lifespan (Cui et al. 2019). A connection between pronounced cellular senescence and the short lifetime of *N. furzeri* is evident, as the long‐lived zebrafish shows significantly lower expression of SA‐β Gal than killifish (Schöfer et al. 2024). Here, we have found that cellular senescence is activated at the boundaries of the IFR, and we hypothesize that the extent of senescent cells may act as a buffering system to maintain tissue integrity between the opposing effects of cell proliferation and cell death. Thus, cellular senescence appears to serve multiple mechanisms that culminate in a tissue protective function in the mucosal lining.

### 3.3 Is There a Fixed ISC Pool in *N. furzeri* Intestinal Epithelia?

We were able to show that ISCs occur in clusters and that the size of the clusters and the number of ISCs per cluster remain constant during lifetime. However, we found that the cell cycle time of ISCs slows down with age, which can be compared to stem cell exhaustion observed in mammals. We also showed that intestinal stem cells have a clear preferential location in the interfold region (IFR), and that these ISC‐containing areas form zones that are separated by areas where no ISCs were detected, although all IFRs contain cycling cells. Within the zones containing stem cells, ISCs were found to be present in clusters. A cluster of ISCs is intermingled and surrounded by dividing cells, presumably belonging to the TA population. Mechanistically, the expansion of these cells would inevitably lead to the fragmentation of ISC clusters into smaller units over time. We performed serial sectioning to rule out a possible spatial bias by evaluating ISC distribution on single sections (Figure S2e1–5). However, ISC clusters were preferentially found within the same zones in adjacent sections, ruling out such a bias. Also, we did not observe ISC clusters becoming smaller or more fragmented with age. We also observed a shift in the localization of ISC clusters towards the folds with age. In the mechanistic scenario, the spreading of ISCs could be explained as a result of the mixing of ISCs with rapidly dividing cells of the TA population, which may force the clusters away from the IFR region. Shifting ISCs towards the folds may represent a mechanism to counteract reduced cell turnover, but it remains difficult to explain how sufficient epithelial tissue homeostasis can be achieved throughout the intestine of old animals.

We therefore propose a dynamic model of ISC induction that maintains tissue integrity. We postulate that the pattern observed in sections represents a snapshot in time. Consequently, we predict that ISCs do not represent a distinct cell population as in mammals, but rather that dividing cells that can be considered equivalent to the mammalian transient amplifying (TA) cells (i.e., the PCNA‐positive cells) or perhaps even differentiated epithelial cells are recruited to become ISCs when required for tissue preservation (“on demand”). The underlying signaling mechanisms and pathways are currently unknown, but we suggest that physical tensions or shear forces in the mucosa may be involved in this process, possibly via the secretion of stem cell niche factors by the lamina propria. A candidate cell for producing these factors might be myofibroblasts that were identified in this study. Indeed, comparable data exist for myofibroblasts in the mammalian intestine, where they have been shown to play an important role in epithelial regeneration and differentiation, helping to define the stem cell niche of the intestinal epithelium (McCarthy et al. 2020; McCarthy et al. 2023; Roulis and Flavell 2016; Xiang et al. 2023; Takashima et al. 2013). In this dynamic “on demand” model, the shift in ISC cluster localization could be attributed to the increased demand for cell renewal in old age, when ISC cycle time increases. It should be noted that the intestines of newborn mice are devoid of crypts, which only develop a few days later (Sumigray et al. 2018). *Lgr5*‐positive cells, which are indicative of mammalian ISCs, were abundant between the villi prior to crypt formation. However, after crypt formation, these cells become less abundant and are restricted to the crypt base (Shyer et al. 2015). The distribution pattern of ISCs in newborn mice shows similarities to the situation in killifish, although a cluster‐like distribution of ISCs prior to crypt formation has not been reported. In adult mammals, the reprogramming of differentiated cells to replenish the ISC pool has been reported during regeneration of damaged ISCs (de Sousa and de Sauvage 2019; Murata et al. 2020), a process driven by niche factors (Metcalfe et al. 2014). There is a lack of consensus in the field regarding the extent to which plasticity and dedifferentiation contribute to the ISC pool in the normal mammalian intestine (Guiu et al. 2019; Ramadan et al. 2022). Given the embryology and plasticity of mammalian ISCs, our proposed model of permanent ISC recruitment rather than maintenance of a permanent ISC population in killifish may represent an evolutionarily ancient state.

### 3.4 Fasting Attenuates Age‐Dependent Changes in Gene Expression

We applied a late‐onset intermittent fasting (IF) protocol starting from adult animals (10 weeks), whereby one cohort received daily excess food (*ad libitum*; AL) and the other cohort received food every other day (IF); that is, complete alternate fasting days. Global expression of genes involved in food uptake revealed many age‐dependent DEGs and a mitigating effect on the age‐dependent gene expression in the IF group. A general reduction in age‐related diseases and an increase in longevity in killifish undergoing dietary restriction has previously been demonstrated in male but not in female *N. furzeri* (McKay et al. 2022). An increase in longevity has also been observed in our facility using the identical dietary regimen (personal communication O. Pusch). Here, we demonstrate that the beneficial effects observed for the whole organism are reflected in a specific organ, the gut tube. Our study was conducted with male animals only. In a follow‐up study, it will be interesting to see if sex‐dependent differences exist.

An intriguing finding was the age‐dependent alteration in the expression of circadian rhythm regulators. Circadian rhythmicity is regulated not only at the level of the organism by the brain but also peripherally in all organs by peripheral clock genes. The disruption of circadian rhythm with age does not necessarily have a direct relationship to gene expression levels, as shown in a study of *bmal1* in the killifish pineal gland, where cyclic expression amplitudes were altered, rather than gene expression levels (Lee et al. 2021). However, this finding does not exclude the possibility that altered overall gene expression levels may influence circadian rhythms in peripheral organs. Indeed, age‐related decline in clock gene expression in different organs, such as *bmal1*, has been shown in male mice (Wolff et al. 2023) and in killifish (Almaida‐Pagan et al. 2018; Barth et al. 2021; Lee et al. 2021). The gut holds a key position as the organ responsible for nutrient uptake and it is known that glucose, fatty acid, and cholesterol metabolism and nutrient‐sensing pathways, that is, pathways that are regulated by nutrient availability, are under circadian control (Takahashi 2017). Disruption of the circadian clock has been associated with adverse effects on health status and longevity. The expression of nutrient‐sensing genes fluctuates throughout the day (Acosta‐Rodriguez et al. 2021), suggesting a potential link between circadian rhythm and food consumption. The highly significant age‐dependent alteration of clock gene expression, coupled with the observed reversal in the IF fish group, underlines this link and highlights the beneficial effect of fasting on daily rhythmicity in killifish.

The present study employs the intermittent fasting (IF) protocol with alternating fasting and *ad libitum* (AL) feeding. A striking effect was the observation that the gene expression profile of the old fish IF group is more similar to that of the adult group than to that of the old fish AL cohort. Week 20 IF animals showed a gene expression pattern comparable to that of adult animals, which suggests a protective effect of fasting on several biological functions. It should be noted that, with the experimental design used, we cannot determine whether IF prevents age‐related upregulation or downregulation or if it rather delays it. To clarify this point, a study would be required with at least one more age sampling point.

The results of ongoing studies will show whether other organs of the killifish display similar changes in gene expression profiles during aging and whether the IF regimen has a similar protective effect on gene expression in these organs. A limitation of the chosen IF approach is that it is not possible to disentangle the effects of calorie reduction in the IF group compared to the AL group, or to establish whether fasting per se is responsible for the observed effects in gene expression. This is because the IF strategy does not allow us to measure total calorie uptake in the two cohorts. Furthermore, we cannot rule out the possibility of adverse health effects of dietary restriction, although we did not observe significant behavioral differences between the two groups. Additionally, the lifespan of the IF animals increases significantly over that of the AL group (personal communication O. Pusch). Regarding the feeding behavior of IF animals, there are indications of partial compensation of consumed larvae, suggesting that it is fasting itself, rather than calorie restriction, that maintains a gene expression profile of adult fish in old animals. This would be consistent with studies in male mice that suggest it is fasting itself, more precisely the delay between the feedings, that has a dominant effect on lifespan extension and not calorie intake and diet composition (Mitchell et al. 2019).

Our study demonstrates that killifish and mammals share basic anatomical, histological, and tissue regenerative traits. Examples include the mucosal architecture showing surface enlargements, enterocytes with a well‐developed brush border, the presence of goblet cells, and the decline in intestinal stem cell activity with age. However, other features differ between killifish and mammals. For instance, fish lack a stomach, crypts, and, apparently, a dedicated stem cell population. Fasting has been shown to improve ISC function during aging in various aging model systems (Fontana and Partridge 2015). A recent study reported that IF maintained the intestinal epithelial cell type equilibrium by maintaining mTORC1 signaling in *Drosophila* (Mattila et al. 2024). Dietary restriction in killifish has been shown to slow down aging processes and extend life expectancy (McKay et al. 2022; Terzibasi et al. 2009). As demonstrated by Yilmaz et al. (2012), fasting has been shown to improve intestinal stem cell (ISC) activity via Paneth cells in aged mice. Also, in aging mice, IF has been shown to preserve homeostasis of the epithelial lining (Gebert et al. 2020) and to augment ISC activity by inducing a fatty acid oxidation program (Mihaylova et al. 2018). In humans, calorie restriction has been shown to be beneficial for mitochondrial stability by reducing reactive oxygen species due to the reduced metabolic rate of cells (Das et al. 2023; Hastings et al. 2024). Thus, the maintenance of adult gene expression in aged killifish by IF, and presumably the maintenance of ISC function, is consistent with the findings of studies conducted in mammals. Together, considering the similarities and differences between killifish and mammals, the results of this study extend beyond killifish and may be relevant to other vertebrate clades.

## 4 Methods

### 4.1 Animal Husbandry and Processing

*N. furzeri* (strain GRZ) was maintained at a facility at the Center for Anatomy and Cell Biology, Medical University of Vienna under standard conditions as described in (Kabiljo et al. 2019). In brief, killifish were kept under a 12 h dark–light cycle at 28°C. Animals were fed a diet of nauplia larvae for newborns and *Chironomus* larvae for older stages. Animals were fed twice daily with an excess of *Chironomus* larvae. Using these conditions, the maximum lifespan of our strain is 20 weeks (Zupkovitz et al. 2021), which is defined as the point at which < 10% of a cohort survives. Only male animals were used in this study.

### 4.2 Dietary Regimen

A late‐onset dietary intervention was applied: male fish were transferred to single housing in 2.8 L tanks when showing the first signs of sexual maturation (around Week 4–5). Ten‐week‐old animals (adult age) were separated into three groups: one group of animals was immediately sacrificed, and two cohorts were sacrificed at 20 weeks of age (old age). One cohort continued to receive excess food twice daily (*ad libitum*, AL), while the other cohort received food every other day (known as the “alternate‐day fasting” (ADF) or “every‐other‐day fasting” regimen) for 10 weeks. ADF is a type of intermittent fasting (IF) regimen; for simplicity, we refer to this type of dietary restriction as “IF.” The feeding times and the amount of food per animal were identical for the AL and IF cohorts, that is, when the latter were fed.

### 4.3 Histology

Killifish was processed *in toto* until 5 weeks of age; in older animals, the gut tube was isolated. For post‐mortem analysis, old fish that were alive in the morning and found dead later that day were used. The samples were fixed overnight with 4% paraformaldehyde (PFA), washed with PBS, and embedded either in cryomolds with O.C.T. compound (Tissue‐Tek, Sakura Finetek) or in paraffin using a standard tissue embedding procedure. Paraffin sections of human intestine were from archival material.

### 4.4 High‐Resolution Episcopic Imaging (HREM) of Total Fish

HREM procedure of an adolescent fish (5 weeks of age) followed standard protocols (Geyer et al. 2017). In short, the specimen was fixed in Bouin's solution for 24 h, washed in phosphate‐buffered saline (PBS), dehydrated in a graded series of ethanol, and subsequently embedded in methacrylate resin (JB‐4, Polysciences) containing eosin (0.4 g/100 mL). The blocks were allowed to polymerize at room temperature, followed by baking at 90°C for 1 day before subjecting them to HREM volume data production. Digital images of the block surface were recorded on a conventional HREM apparatus (Indigo Scientific Ltd.), resulting in a stacked data series with isotropic voxels of 3 μm side length. The HREM data were analyzed, and intestines were manually segmented with the software package Amira 7.0 (ThermoFisher Scientific).

### 4.5 Electron Microscopy

Samples were fixed with 4% glutaraldehyde in 0.1 M phosphate buffer overnight, post‐fixed with 1% aqueous OsO_4_, and embedded in Epon 812 according to standard procedures. Semi‐thin and ultra‐thin sections were cut using a Reichert Ultracut S microtome and stained with toluidine blue (semi‐thin) or were contrasted with uranyl acetate and lead citrate (ultra‐thin).

### 4.6 Brillouin Light Scattering (BLS) Microscopy Measurements

Gut tubes of three animals (age 9 weeks) were isolated, put in PBS and immediately transferred to the BLS measurements, where samples were removed from the PBS and placed in a petri dish below the objective. Measurements were immediately performed to avoid significant dehydration of the sample at three different regions of the fish gut: the intestinal bulb (anterior intestine; AI), middle intestine (MI), and posterior intestine (PI). The confocal optical setup consisted of an upright excitation/detection arm with a 20× lens NA = 0.42 (Mitutoyo, back aperture overfilled) to which excitation light (532 nm CW single longitudinal mode laser) was coupled via a polarizing beam splitter. This was followed by a quarter waveplate (such that the sample was probed using circularly polarized light). The back scattered light (which consisted of BLS scattering from predominantly longitudinal phonon modes) was focused through a pinhole of approximately 1 Airy Unit in size, before entering the spectrometer. This configuration gave a spatial resolution of approximately 0.8 μm laterally and approximately twice this axially. The spectrometer consisted of a 3 + 3‐pass tandem Fabry‐Pérot interferometer (Table Stable Ltd., TFP HD2) with a finesse > 100. The mirror spacing of the interferometer was 11 mm and the scanning amplitude 450 nm (corresponding to a spectral range of 24 GHz). The signal (detected by a Hamamatsu Photonics K.K detector) was processed by a multichannel analyzer with 512 channels and analyzed in the GHOST 7.00 software. The acquired spectra were fitted with Lorentzian functions together with an exponential function accounting for the tail from the strong Rayleigh scattering using a homemade Matlab (Mathworks) script to obtain the Brillouin frequency shift $\left(\nu_{b}\right)$ and linewidth $\left(\Gamma_{b}\right)$. Because the refractive index and mass density of the *N. furzeri* gut tissue is not known, we used the mechanical loss tangent (tan(δ)) = $\Gamma_{b}/\nu_{b}$, which is independent of density and refractive index to ascertain changes in the mechanical properties (Elsayad 2021). The mechanical loss tangent, essentially measures how efficiently a material dissipates mechanical forces. When tan(*δ*) equals 0, it signifies complete elasticity of the material, whereas a larger value indicates that energy will be lost during mechanical perturbations.

### 4.7 Immunostaining

Paraffin or cryo sections were treated with a heat‐induced epitope antigen retrieval (HIER; steaming with 10 mM citrate buffer pH = 6.0 for 30 min) followed by 0.5% Triton X‐100 and were incubated with the following primary antibodies: mouse anti‐PCNA antibody (sc‐56, Santa Cruz Biotechnology; RRID:AB_628110), rabbit alpha Smooth Muscle Actin antibody (GTX100034, GeneTex; RRID:AB_1240408), rabbit ribosomal protein L7a antibody (2403, Cell Signaling; RRID:AB_2182061) overnight at 4°C and detected by immunofluorescence staining (PCNA, alpha Smooth Muscle Actin) with goat anti‐mouse Alexa488 antibody (Invitrogen) or goat anti‐rabbit Alexa488 antibody (Invitrogen). Autofluorescence quenching was performed using TrueView (Vector Laboratories) prior to counterstaining with DAPI (0.1 μg/mL). Ribosomal protein L7a was detected by alkaline phosphatase conjugated—anti‐rabbit antibody (A3687, Sigma‐Aldrich) using the NBT/BCIP developing system (Roche). For the detection of goblet cells on sections, FITC‐conjugated WGA (wheat germ agglutinin; V1028; Vector) was used 1:100 in PBS.

### 4.8 Apoptosis Detection TUNEL

Deparaffinized and rehydrated paraffin sections were treated with 20 μg/mL Proteinase K for 20 min at 37°C. TUNEL assay was performed using the In Situ Cell Death Detection Kit, Fluorescein (11684795910 Roche/Sigma‐Aldrich) according to the manufacturer's instructions. Samples were counterstained with DAPI (0.1 μg/mL) at room temperature and mounted with Citifluor (EMS).

### 4.9 SA‐βGal (SABG) Staining for Cellular Senescence

Cryosections were treated with a commercially available staining kit (9860, Cell Signaling) according to the manufacturer's instructions. Slides were washed in distilled water and in PBS and were incubated with ß‐galactosidase staining solution pH = 6.0 in a sealed chamber overnight. After washing the slides with PBS, staining with DAPI (0.1 μg/mL) for 6 min was performed. Cryosections were mounted in Citifluor (EMS).

### 4.10 Pulsed Injections of Labeled Precursors to Detect Label‐Retaining Cells (Stem Cells)

Experimentation with animals was approved by the Austrian Federal Ministry of Education, Science and Research (GZ: BMWFW‐66.009/0031‐V/3b/2019). Adolescent (5 weeks), adult (10–12 weeks) and old age (18–29 weeks) male fish were anesthetized with 100 mg/L MS‐222 (tricaine, Sigma E10521). Fish were intraperitoneally injected with volumes of 10 μL (adolescence) and 15 μL (adult and old age) of 10 mM BrdU and 100 μM EdU in water. BrdU was administered twice, separated by 2 h, followed by a 6‐day interval before the EdU injection. After injections, animals were placed into their respective tanks and sacrificed 1 day after the EdU administration using a lethal dose of MS‐222 (400 mg/L).

### 4.11 Detection of BrdU and EdU on Paraffin Sections

Deparaffinized and rehydrated sections were treated with 0.5% Triton for 10 min, followed by incubation with 2 M HCl for 60 min at 37°C and with 0.4% pepsin for 30 min at 37°C. EdU detection was performed using the Click‐iT EdU imaging Kit, Alexa Fluor 647 dye (C10340 Invitrogen) according to the manufacturer's instructions. BrdU was detected with a mouse anti‐BrdU antibody (1170376 Roche) and with the secondary anti‐mouse Alexa 488 antibody. In some experiments, autofluorescence quenching was performed using the TrueView (Vector) kit prior to counterstaining with DAPI. Sections were mounted with Vectashield Antifade Mounting Medium (Vector) or, in the case without quenching, in Citifluor (EMS) and were evaluated with an automated slide scanning system. A cluster of ISCs was defined as a group of cells containing two or more ISCs separated by no more than 10 DAPI‐positive nuclei apart. Cluster sizes (i.e., all cells in the cluster) and the numbers of ISCs per cluster were counted. A technical bias was excluded by serially sectioning the intestines of injected animals (Figure S2e1–5).

### 4.12 Peroxidase Detection in Gut Tubes

Newborn (4 days old) fish were incubated in tank water containing 10 mg/mL peroxidase for 2 h, sacrificed, and immediately fixed in 4% PFA and washed with PBS. The gut tubes were isolated, digested with 0.1 mg/mL collagenase for 20 min, washed with PBS, and incubated with DAB (3,3′‐diaminobenzidine). The reaction was stopped in PBS after visual inspection.

### 4.13 In Situ Hybridization

In situ hybridization probes for *apoa1* (antisense and for control purposes sense probes) were generated with PCR amplification using T3 and T7 specific primers (Table S2). Probes were in vitro transcribed and labeled with digoxigenin (DIG RNA Labeling Mix, 11277073910, Roche, Sigma‐Aldrich) using the Maxiscript T7/T3 Transcription Kit (AM1324, Invitrogen) according to the manufacturer's instructions. In situ hybridization was performed as previously described (Murko et al. 2010). For detection of rRNA, a clone containing the EcoRI fragment A consisting of the 5′end of 18S, 5.8S, and most of 28S rDNA from humans was used (Sylvester et al. 1986); ISH with no probes served as a control for the rRNA experiment. Clones were labeled with digoxigenin using the DIG‐Nick Translation Mix (Roche) according to the manufacturer's protocol. For ISH, 4% PFA fixed samples were dehydrated and embedded in paraffin using a standard tissue embedding procedure. Deparaffinized, rehydrated, and post fixed (4% PFA) 5 μm sections were incubated with 0.2 M HCl for 10 min and digested with proteinase K. After dehydration, air‐dried sections were incubated with the corresponding probes in the hybridization mix containing 2 × SSC, 50% formamide, 10% dextran sulfate, 0.02% SDS, 100 ng/μL, yeast t‐RNA, and 1 × Denhardt's solution overnight at 55°C in a humidified chamber. Washing steps were performed at 42°C with 2 × SSC for 10 min three times and with 2 × SSC at room temperature for 10 min. After a blocking step with 2% FCS for 30 min, probes were detected with an alkaline phosphatase conjugated sheep anti‐digoxigenin antibody (11093274910, Roche, Sigma‐Aldrich; RRID:AB_2313640) using the NBT/BCIP developing system (Roche).

### 4.14 Imaging

Large field‐of‐view images were acquired with a 40× NA 1.3 dry lens on an Evident VS120 automated slide scanning system (Evident Tokyo, Japan). Images were stored in the proprietary vsi format. Images were viewed with the software VS Desktop (Evident) and suitable areas were transferred to image analysis software Fiji (Schindelin et al. 2012) for measurements. High‐power magnifications and transmitted light images were acquired with a 100× NA 1.4 immersion oil lens on a Nikon Eclipse 800 microscope (Nikon, Tokyo Japan) and images were stored in jpeg or tif file formats. Ultrastructural images were taken with a FEI Tecnai G2 20 TEM (ThermoFisher) and saved in tif format. All image data were quantified with Fiji software using area and mean gray value measurements. The paired Student's *t*‐test with a two‐tailed distribution was used for statistical analysis of image data.

### 4.15 RT‐PCR and qRT‐PCR

For the detection of gene expression in intestinal segments (Figure 2c; d1,2), intestines from six adult, AL‐fed animals (11 weeks old) were cut as indicated in Figure S1b1–3. Identical segments of three animals each were pooled and processed in two experimental approaches. For age‐dependent expression of selected stemness genes (Figure S2f1–5) intestines of 38 pooled 4 days old animals and separately four 5 week old animals, three 12 weeks old animals, and four 18 weeks old animals were used. Tissue samples were homogenized in TRI Reagent (T9424, Sigma) and the Quiagen tissue lyser. After treatment with 1‐bromo‐3‐chloropropane (B9673, Sigma) RNA was isolated by a standard isopropanol precipitation. RNA was purified and concentrated by the Zymo RNA Clean and Concentrator Kit (R1013, Zymo Research) including a DNase I incubation step according to the manufacturer's instructions. The pellet was dissolved in nuclease free water, and RNA concentration was measured with DS‐11 (Denovix). 400 ng (RT‐PCR) or 1 μg (qRT‐PCR) total RNA was reverse transcribed into cDNA using iScript cDNA Synthesis Kit (1708890, Bio‐Rad) or LunaScript RT SuperMix Kit (E3010, New England Biolabs). PCR products were generated with gene specific primers (Microsynth) as listed in Table S2 using Taq PCR Master Mix Kit (201443, Qiagen) and selected purified PCR products were confirmed by standard DNA sequencing procedures (Microsynth). Sequence retrieval and primer design (Primer BLAST) was performed in NCBI databases. Real‐time PCR (qPCR) was done using the Blue S'Green qPCR Kit (331416, Biozym) on a CFX384 qPCR machine (BioRad). Gene expression was normalized to the *tbp* (TATA‐binding protein) housekeeping gene (Baumgart et al. 2014). qRT‐PCR investigations were performed in three technical replicates in three independent experiments. Data distribution was tested with the Kolmogorov–Smirnov test; significant differences were tested either with the non‐parametric one‐way Kruskal–Wallis ANOVA (non‐normal distribution) followed by Dunn's test for pairwise comparison or by ANOVA (normal distribution) followed by Tukey's test for pairwise comparison, both for *α* = 0.05, using GraphPad Prism software.

### 4.16 Differentially Expressed Genes in Whole Intestines (RNASeq)

For age‐ and diet‐dependent studies, a late‐onset dietary restriction approach was chosen. Five animals per cohort (*n* = 5) were euthanized with MS‐222, small intestines were dissected, and snap‐frozen in liquid N_2_. Total RNA was extracted in TRI‐Reagent (T9424, Sigma) using Qiagen TissueLyser LT. Phase separation was performed in Phasemaker Tubes (Invitrogen) by the addition of 1‐Bromo‐3‐Chloropropane (B9673, Sigma), vigorous shaking, incubation for 3 min at room temperature, and centrifugation at 12,000 rpm at 4°C for 15 min. The upper phase was mixed with 70% ethanol, and subsequent RNA purification, including a DNase I digestion step, was accomplished using the ReliaPrep RNA Tissue Miniprep System (Z6111, Promega) according to the manufacturer's instructions. Quality of RNA was assessed using a 4200 TapeStation system (Agilent). Libraries were prepared from 1 μg of isolated total RNA. Enrichment of mRNA was performed via oligo dT‐based mRNA isolation by using NEBNext Poly(A) mRNA Magnetic Isolation Module (E7490L; NEB). For cDNA library preparation, adapter ligation and PCR enrichment steps, the NEBNext Ultra II Directional RNA Library Prep Kit for Illumina (E7760L, NEB) in combination with NEBNext Multiplex Oligos for Illumina (Dual Index Primers Set1) (E7600S, NEB) was used according to the manufacturer's instructions (adaptor dilution: fivefold, 8 amplification cycles). Purification steps were performed with Beckman Coulter AMPure XP beads. Library quality was assessed using the 4200 TapeStation system.

Sequencing was performed at CEITEC (Bioinformatics Core Facility, Centre for Molecular Medicine, Masaryk University, Brno, Czech Republic). Samples were sequenced using paired‐end 75 bp reads and raw reads were quality checked (FastQC; (https://www.bioinformatics.babraham.ac.uk/projects/fastqc/)), preprocessed (Trimmomatic; (Bolger et al. 2014)) and mapped (STAR; (Dobin et al. 2013)) to the reference genome (Nfu_20140520) with corresponding gene annotation (ENSEMBL release 108; (Harrison et al. 2024)). Overall sample quality was assessed according to rRNA content estimate (fastq_screen), read duplication rate (dupRadar, Picard tools), sequenced (targeted) regions (RSeQC, Picard tools), 5′/3′ coverage bias (Picard tools), expressed gene biotypes (featureCounts) and library strandness (RSeQC). One sample of the IF group failed quality assessment ending up with *n* = 5 for 10 weeks and 20 weeks AL and *n* = 4 for 20 weeks IF. Differentially expressed genes (DEGs) in each intestinal tube were identified using edgeR (Robinson et al. 2010) and DESeq2 (Love et al. 2014) and sorted according to an adjusted *p*‐value of < 0.05 and an absolute log2fold change value of 1. Venn diagram (Figure 4b) was generated with an online tool from Ghent university (https://bioinformatics.psb.ugent.be/webtools/Venn/) using the significant DEGs identified by DESeq2. Gene set enrichment in Figure 4c1–6 was done on the significant DEGs identified by DESeq2 using ShinyGO 0.80 from South Dakota State University (http://bioinformatics.sdstate.edu/go80/; (Ge et al. 2020)) with *N. furzeri* ENSEMBL IDs. For GO‐terms “all available gene sets” was selected. The heatmaps in Figures 5 and 6; Figures S4 and S5 were compiled by manually selecting factors from the literature that are known to be involved in specific functions or structural elements. These genes relate mostly to fish and mammalian species. The heatmaps were created with Excel spreadsheet comparing normalized counts obtained by DESeq2 analysis. Trimmed mean of *M*‐values (TMM) were obtained using EdgeR.

## Author Contributions

**Christian Schöfer**, **Klara Weipoltshammer**, **Oliver Pusch:** conceptualization. **Christian Schöfer**, **Oliver Pusch**, **Stefan H. Geyer**, **Elmar E. Ebner**, **Wolfgang J. Weninger**, **Kareem Elsayad**, **Sabine Lagger, Sylvia Laffer:** methodology. **Sylvia Laffer**, **Michael Kothmayer**, **Philipp Widmayer**, **Elmar E. Ebner**, **Fehima Ugarak**, **Stefan H. Geyer**, **Christian Schöfer:** investigation. **Christian Schöfer**, **Sylvia Laffer**, **Sabine Lagger**, **Michael Kothmayer**, **Philipp Widmayer**, **Fehima Ugarak**, **Kareem Elsayad**, **David Martin:** data analysis. **Christian Schöfer:** funding and supervision. **Christian Schöfer**, **Klara Weipoltshammer:** writing. **Christian Schöfer**, **Klara Weipoltshammer:** manuscript editing; wrote the manuscript with contributions from all other authors. All authors read the final version.

## Ethics Statement

Animal experimentation was approved by the Austrian Federal Ministry of Education, Science and Research (GZ: BMWFW‐66.009/0031‐V/3b/2019); killifish rearing is licensed under BMBWF‐66.009/0130‐V/3b/2018 (Austrian Federal Ministry of Education, Science and Research).

## Conflicts of Interest

The authors declare no conflicts of interest.

## Supporting information


**Figure S1.** Morphology of the killifish gut tube. (a) prominent DAB staining in the PI of isolated gut tubes (a1: sham incubation, a2: peroxidase incubation). (b) stretched gut tube in toto (b1), after histology (b2; HE staining) and the nomenclature applied (b3); stretches indicated with S denote segments used for PCR analysis. (c) morphometry of growth (c4 and Figure 1b) and weight gain of body (c5) and of gut tubes (c3) and morphometry of inner intestinal surface area (c2) over lifetime; linear regression gut versus body length (c1) and body versus gut weight (c6); bars represent mean values, whiskers ± SD. (d) d1: Maier–Kaplan survival curve of the GRZ strain in our facility (curve taken from Zupkovitz et al. 2021) with indicated age stages used in this study (newborn‐4 days; adolescence‐5 weeks, adult 10–13 weeks and old age 18–20 weeks); d2: images of fish at different ages showing overall growth and also age‐related change of body shapes (all males from adolescence onwards). (e) longitudinal section (e1; staining with WGA to show goblet cells [green]) and semi‐thin section (e2) of mouth and pharyngeal cavity (white asterisks) with abundant goblet cells (red asterisk) and intraepithelial glands (red arrow), the latter is lacking in the intestinal lining; white star: intestine with goblet cells, white arrow: mouth. *p < 0.05; **p < 0.01; ***p < 0.001; ns, not significant; Student's paired *t*‐test with a two‐tailed distribution.
**Figure S2.** Morphology of the killifish gut tube and intestinal gene expression. (a) a1: anterior intestine (AI), cross section, longitudinally opened intestine (AI) low (a2) and higher power magnification (a3) showing abundant, irregular folds. (b) the tunicae musculares (dashed lines) enter the incision between MI (right) and PI (left); HE staining. (c) c1 shows low power magnification of pharyngeal roof with pads (one indicated with dashed line); c2 shows higher power magnification of pharyngeal pads with clearly visible arrays of teeth (arrow) and some isolated skeletal muscle fibers extending from pads. (d) histology of a W20 post‐mortem gut tube showing intact mucosa, d1: overview; d2: higher power magnification of boxed area in d1; HE staining. (e) e1‐5 example of a series of five consecutive sections simultaneously stained for detection of BrdU (green) and EdU (red) incorporation (DAPI blue). (f) age‐dependent gene expression (qRT‐PCR) of selected stemness genes (f1‐5) in all four age stages in AL‐fed animals reveal significant decrease of *acat2* expression between adult and old age; *ascl1a, rspo1, rspo3, sox9b* do not shown significant expression changes between adult and old age; bars represent mean values, whiskers ± SD. (g) g1‐3: pie charts show overview of age‐specific expression (AL and IF) of all manually selected genes used in Figures S5 and S6; note the occurrence of considerably fewer significant differentially expressed genes (DEGs) in the comparison of W10 versus W20 IF than in the comparison between W10 versus W20 AL (heatmaps in Figures S4 and S5). *p < 0.05; **p < 0.01; ***p < 0.001; ns, not significant; one‐way Kruskal–Wallis‐ANOVA followed by the Dunn's test.
**Figure S3:** Intestinal gene expression, RNASeq, top 20 differentially expressed genes (DEGs). (a) a1‐3: pairwise comparison of the top 20 DEGs; clustered heatmaps. (b) b1‐3: depicted are the top 20 DEGs for each pair (volcano plots); red dots highlight genes above *p* < 0.05 whereas black dots represent non‐significant genes; *p*adj < 0.05.
**Figure S4:** Gene expression differences of manually selected sets of factors known to be part of cellular structures or biological processes in other organisms. Heatmaps highlight changes in gene expression based on normalized counts obtained by DESeq2; for expression levels (normalized counts) see Figure 5. (a) with the exception of rora, all peripheral circadian clock genes show significant age‐dependent alterations, while old animals that underwent IF show adult levels. (b) genes involved in nutrient absorption show many DEGs in the AL group that display adult values in the IF cohort. (c) chromatin and DNA damage response (DDR) factors demonstrate adult‐like expression of *ctcf* and *hp1* in old fish of the IF group vs. the AL group. (d) factors for mitochondrial functions show adult levels in the old IF cohort in contrast to the AL group significant age‐dependent changes in *timm23*. (e) factors involved in nucleolar functions and ribosome assembly show adult‐like expression levels of *mTOR, npm, and ubf* in the old IF fish group versus the AL group. (f) the expression of tumor suppressor gene *zpf36* and of autophagy‐related *atg3* shows adult expression levels in the old IF group in contrast to AL animals. Color codes assigned to significance levels (sign); significant differential expression was calculated using Student's paired *t*‐test with a two‐tailed distribution.
**Figure S5.** Gene expression differences of manually selected sets of factors known to be part of cellular structures or biological processes in other organisms. Heatmaps highlight changes in gene expression based on normalized counts obtained by DESeq2; for expression levels (normalized counts) see Figure 6 (a) among senescence and longevity markers the expression levels of *glb1, il‐8*, and *foxo1* are retained in the old fish of the IF group vs. the AL group. (b) markers for epithelial cell types that show adult levels in old fish of the IF group in contrast to the AL group include *slc26a3, dll4*, and particularly, *chgb*, and *pYY*. (c) markers for intestinal stem cells (ISCs) and of the stem cell niche that show adult expression in old age animals of the IF‐group as opposed to the AL animals include *aldh1a2, apc, dll4*, and *znrf3*. (d) significant expression changes of factors involved in Wnt and Notch signaling that include retained expression in the old fish of the IF group versus the AL group include *fzd2* and *znfr3*. Color codes assigned to significance levels (sign); significant differential expression was calculated using Student's paired *t*‐test with a two‐tailed distribution.

**Table S1.** Lists of differential gene expression in intestines detected with RNASeq (EdgeR).

**Table S2.** Primer sequences.

**Video S1.** Rotating virtual fish (volume rendered HREM data) with segmented gut tube (turquoise); same data set as for still image in Figure 1a but without D. choledochus.

## Data Availability

The data that supports the findings of this study are available in the Supporting Information of this article. RNASeq data are available at the Gene Expression Omnibus (GEO) under the accession number GSE284196. All other data can be obtained from the corresponding author upon reasonable request.
