# Supplementary figures and images for: Aging Reduces Intestinal Stem Cell Activity in Killifish and Intermittent Fasting Reverses Intestinal Gene Expression Patterns
Source: Aging Cell. 2025 Sep 22;24(11):e70229. doi: 10.1111/acel.70229 (PMC12611280; doi:10.1111/acel.70229)

Suppl. Fig. 1

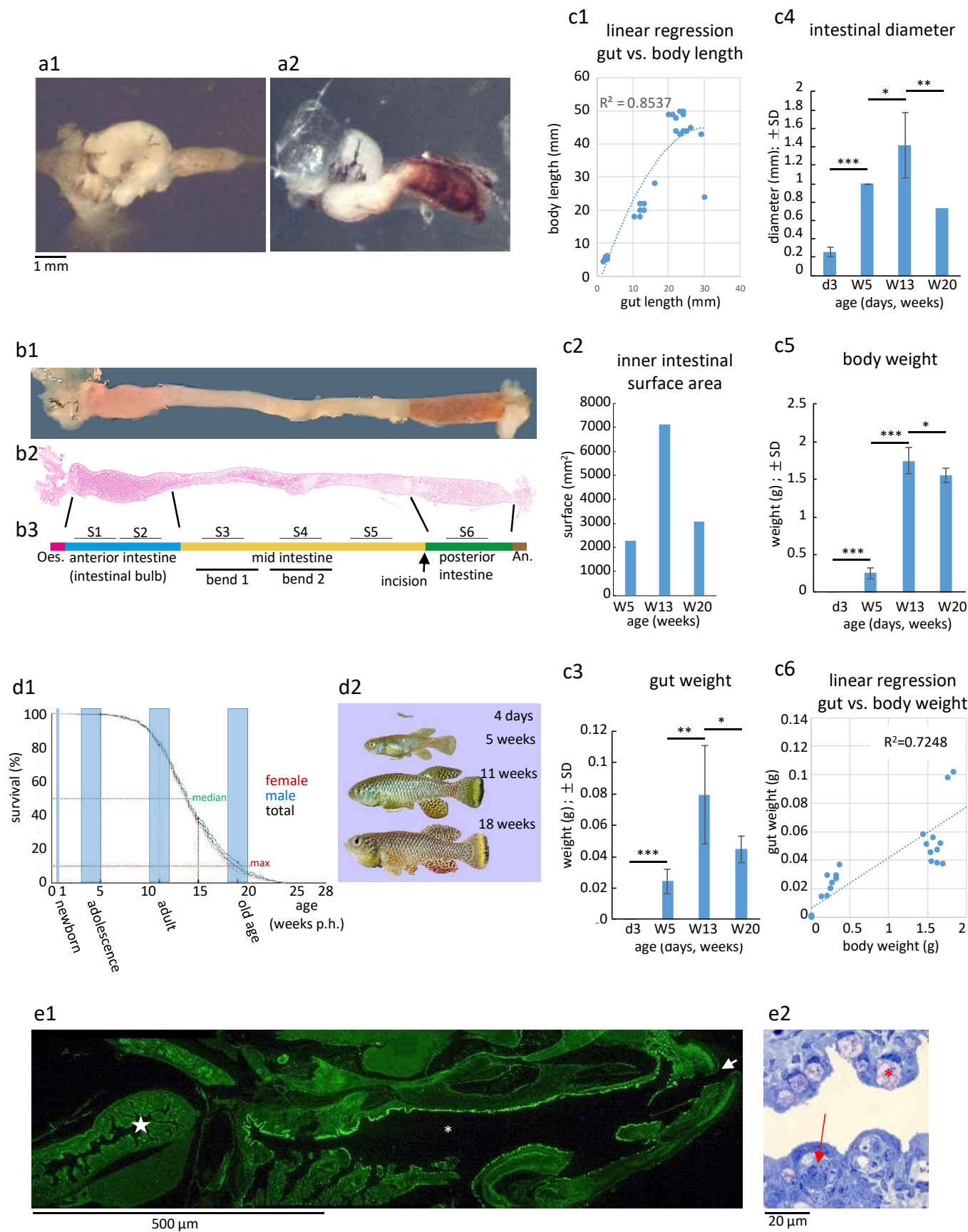

Suppl. Fig. 2

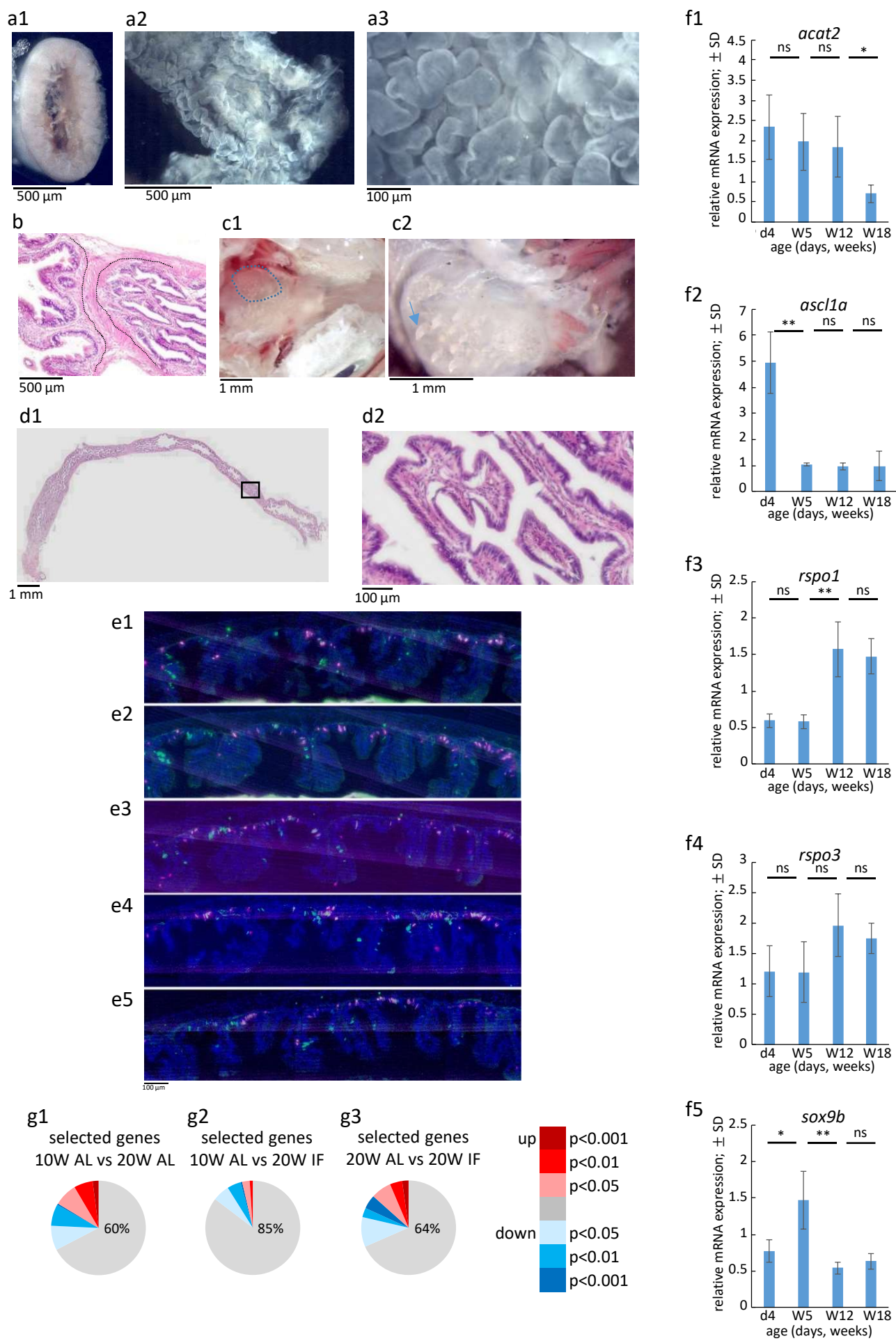

Suppl. Fig. 3

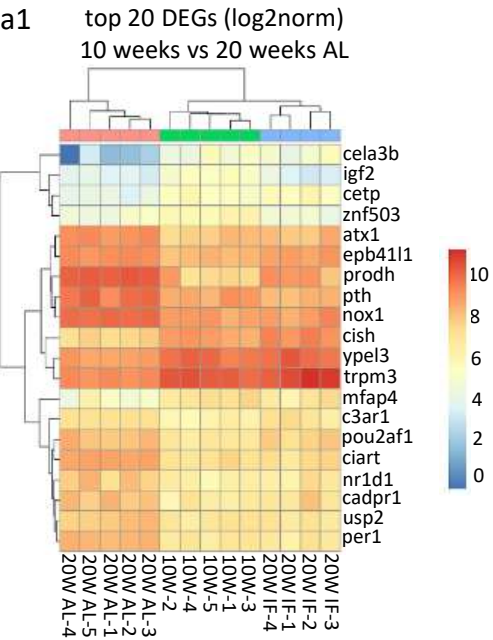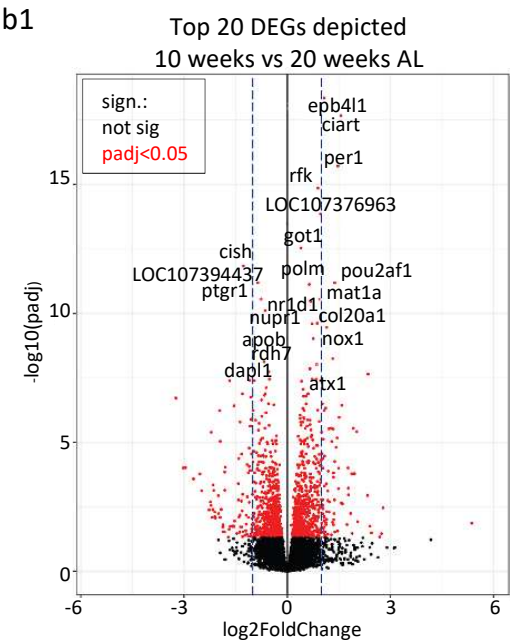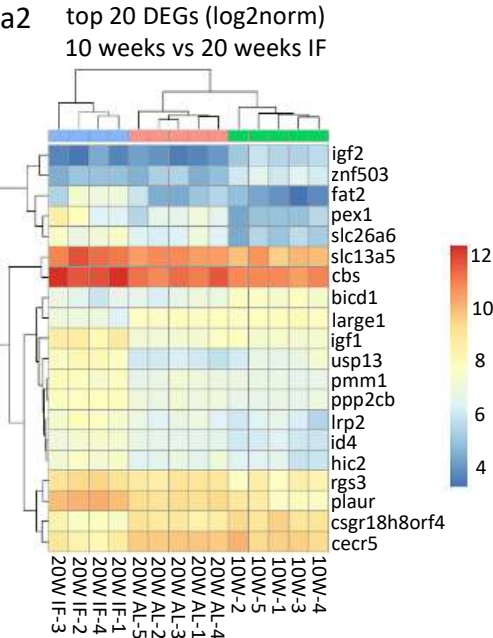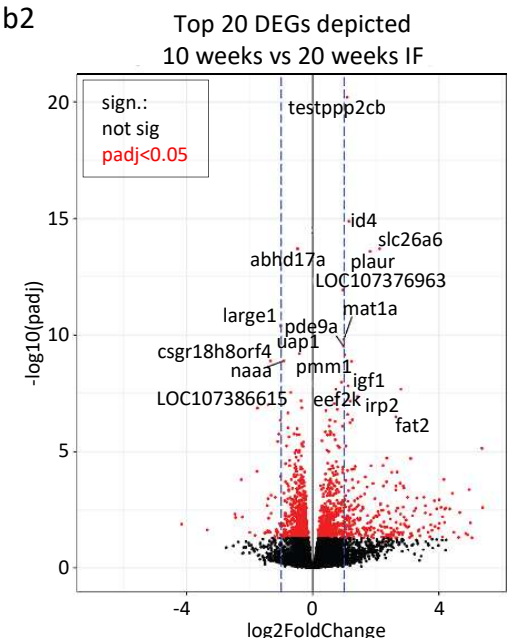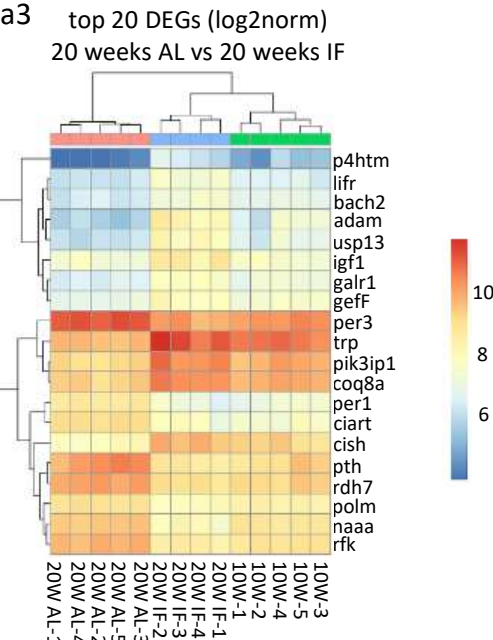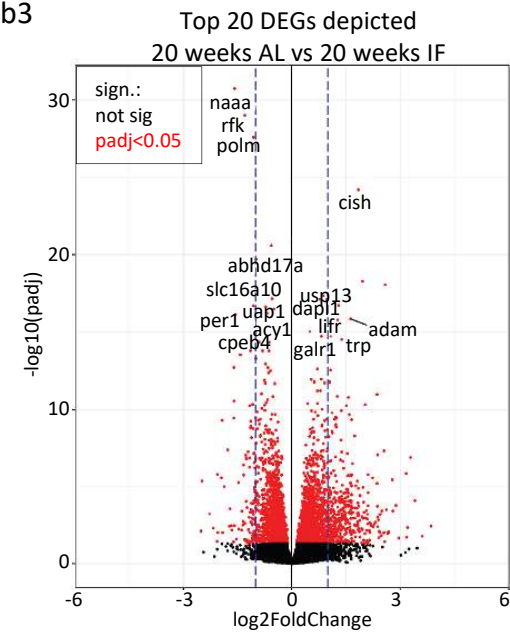

Suppl. Fig. 4

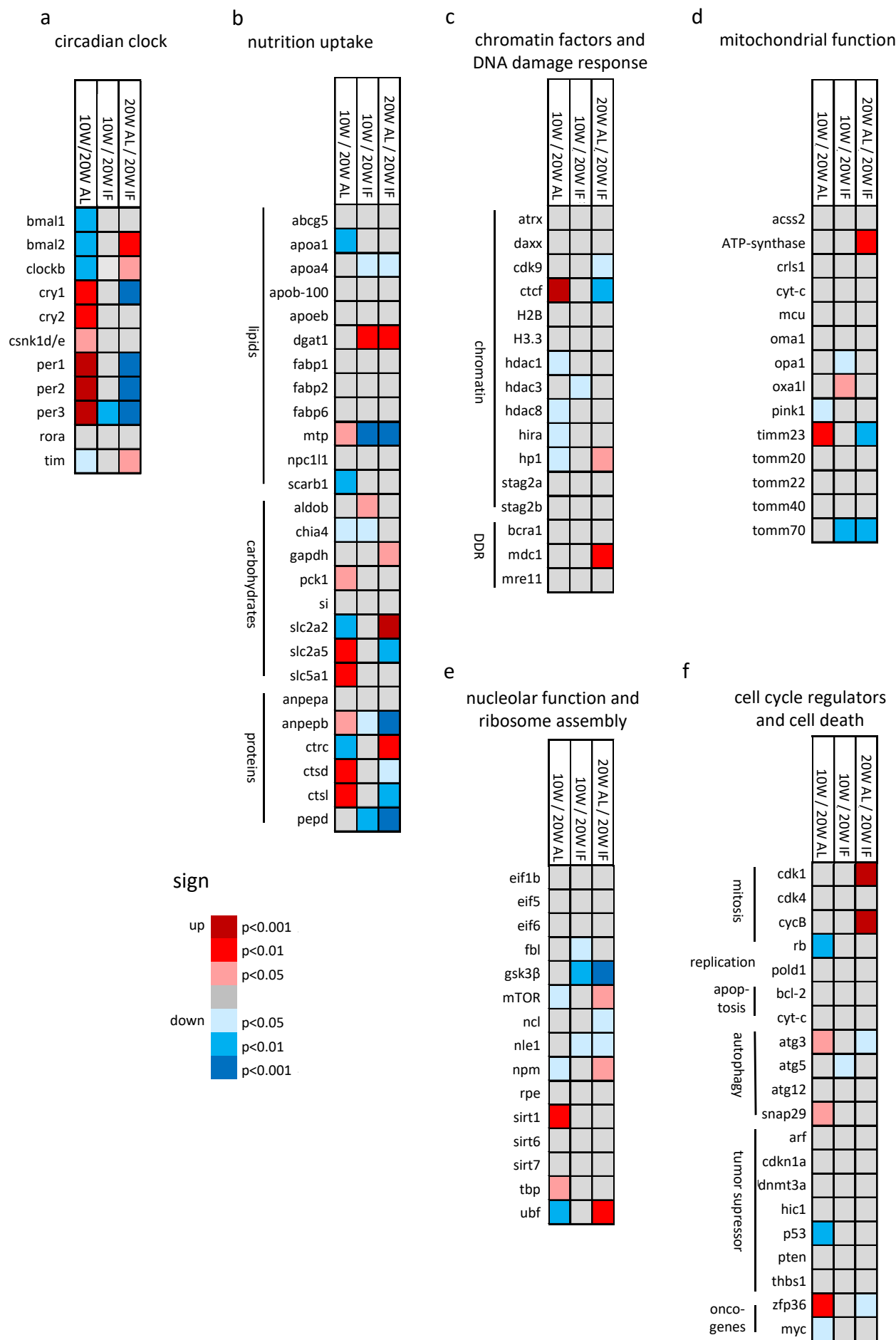

Suppl. Fig. 5

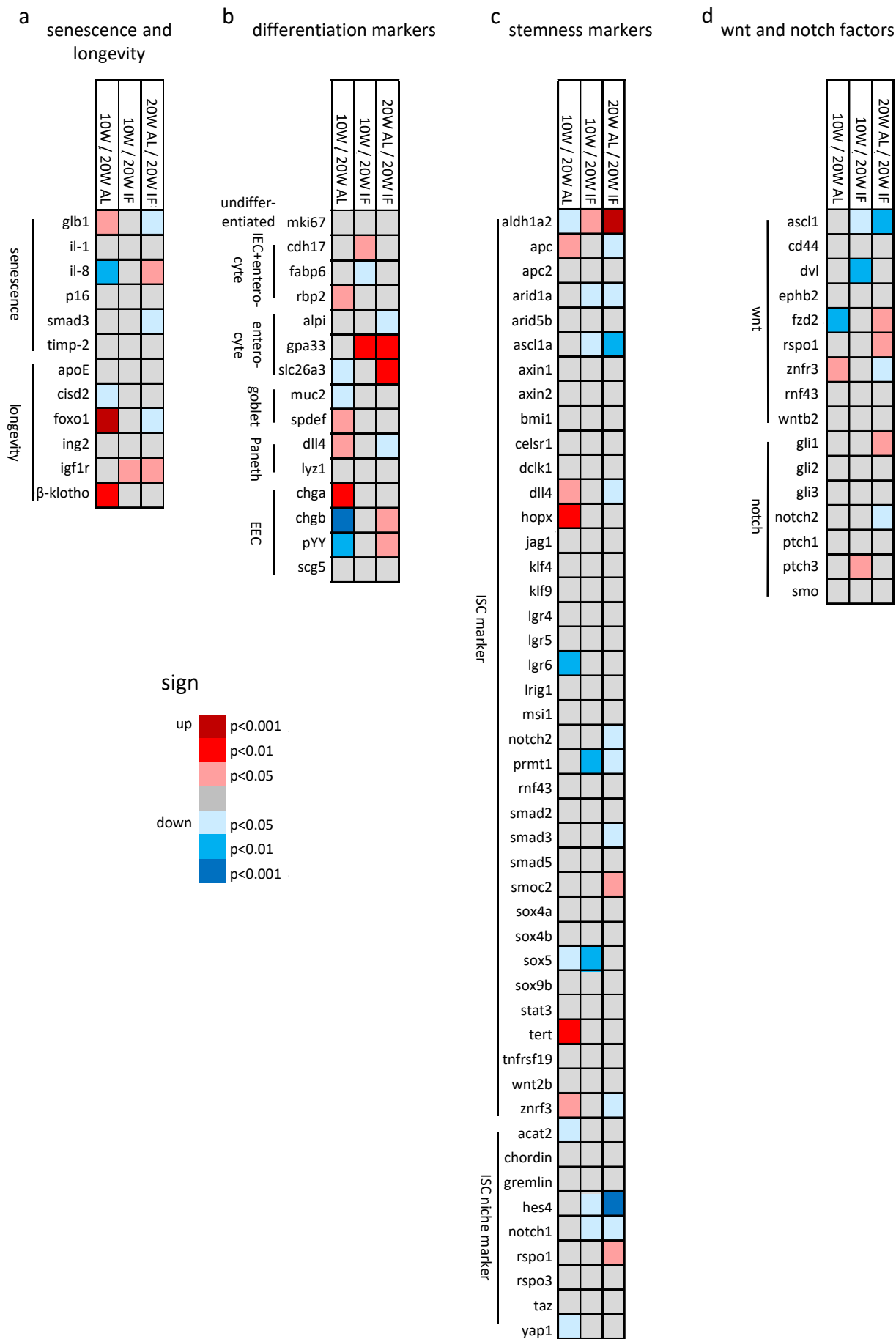

Supplement: Supplementary file 1 — Figure S1. Morphology of the killifish gut tube. (a) prominent DAB staining in the PI of isolated gut tubes (a1: sham incubation, a2: peroxidase incubation). (b) stretched gut tube in toto (b1), after histology (b2; HE staining) and the nomenclature applied (b3); stretches indicated with S denote segments used for PCR analysis. (c) morphometry of growth (c4 and Figure 1b) and weight gain of body (c5) and of gut tubes (c3) and morphometry of inner intestinal surface area (c2) over lifetime; linear regression gut versus body length (c1) and body versus gut weight (c6); bars represent mean values, whiskers ± SD. (d) d1: Maier–Kaplan survival curve of the GRZ strain in our facility (curve taken from Zupkovitz et al. 2021) with indicated age stages used in this study (newborn‐4 days; adolescence‐5 weeks, adult 10–13 weeks and old age 18–20 weeks); d2: images of fish at different ages showing overall growth and also age‐related change of body shapes (all males from adolescence onwards). (e) longitudinal section (e1; staining with WGA to show goblet cells [green]) and semi‐thin section (e2) of mouth and pharyngeal cavity (white asterisks) with abundant goblet cells (red asterisk) and intraepithelial glands (red arrow), the latter is lacking in the intestinal lining; white star: intestine with goblet cells, white arrow: mouth. *p < 0.05; **p < 0.01; ***p < 0.001; ns, not significant; Student's paired t‐test with a two‐tailed distribution. Figure S2. Morphology of the killifish gut tube and intestinal gene expression. (a) a1: anterior intestine (AI), cross section, longitudinally opened intestine (AI) low (a2) and higher power magnification (a3) showing abundant, irregular folds. (b) the tunicae musculares (dashed lines) enter the incision between MI (right) and PI (left); HE staining. (c) c1 shows low power magnification of pharyngeal roof with pads (one indicated with dashed line); c2 shows higher power magnification of pharyngeal pads with clearly visible arrays of teeth [file ACEL-24-e70229-s001.pdf]
