# Supplementary material for: Aging Reduces Intestinal Stem Cell Activity in Killifish and Intermittent Fasting Reverses Intestinal Gene Expression Patterns
Source: Aging Cell. 2025 Sep 22;24(11):e70229. doi: 10.1111/acel.70229 (PMC12611280; doi:10.1111/acel.70229)
Supplement: Supplementary file 3 — Table S2. Primer sequences. [file ACEL-24-e70229-s004.docx]

**Supplementary Table 2**

Primer sequences

| ***exp./gene.*** | **acc.nr.** | **primer name** | **sequence primer (5'-3')** |
| --- | --- | --- | --- |
|  |  |  |  |
| ***qRT-PCR*** |  |  |  |
| *rspo1* | XM_015950062 | rspo1-F | CAAGGCGAGAAGGCAGACAA |
|  |  | rspo1-R | TAGTATCCCACAGGGCAGGA |
|  |  |  |  |
| *rspo3* | XM_015949193 | rspo3-F | GATCTTCTCGCACTGCATGG |
|  |  | rspo3-R | GCATGAATAAGCGTGGCCTG |
|  |  |  |  |
| *ascl1a* | XM_015961432 | ascl1a-F | CGGGAAGTCCAAACAGCTCA |
|  |  | ascl1a-R | CGTTGTTGACCAGTTTCACCC |
|  |  |  |  |
| *sox9b* | XM_015945555 | sox9b-F2 | GCACAACGCAGAGCTCAGTA |
|  |  | sox9b-R2 | CACTCAGGCCGTTCTTGACA |
|  |  |  |  |
| *apoa1* | XM_015950135 | apoa1-Fq | TACGTTGGAAAAGCCACCCA |
|  |  | apoa1-Rq | TCATGTCCTGAGCTCCGATG |
|  |  |  |  |
| *ctsl1* | XM_015960300 | ctsl1-Fq | TGAAGGGGTCCCTGTTCTTG |
|  |  | ctsl1-Rq | CGGTCTTCCTGAACTCCTGAC |
|  |  |  |  |
| *acat2* | XM_015947376 | acat2-F | CGAACCTGTCGTCATCGTTTC |
|  |  | acat2-R | CCCTTGACCTGCTGTTAGGAC |
|  |  |  |  |
| *tbp* |  | tbp-F | CGGTTGGAGGGTTTAGTCCT |
|  |  | tbp-R | GCAAGACGATTCTGGGTTTG |
|  |  |  | Baumgart et al. 2014 |
| ***RT-PCR*** |  |  |  |
|  |  |  |  |
| *apoa1* | XM_015950135 | apoa1 -F | TGCATTCTGGGACTACGTTGG |
|  |  | apoa1 -R | GTCAACATAGGGGGTTGCCA |
|  |  |  |  |
| *fabp6* | XM_015957328 | fabp6-F | CGCTGGAAAATGGGAAGTCG |
|  |  | fabp6-R | CTCGCTGACTTGACGGTAGT |
|  |  |  |  |
| *ctsl1* | XM_015960300 | ctsl1-F | CCTGTTCTTGGAGCCCAACT |
|  |  | ctsl1-R | GTCCCCAGGTATGGGTAGGA |
|  |  |  |  |
| *ctsd* | XM_015954041 | PGA-F | AGCGCTCCCTGAAGTACAAC |
|  |  | PGA-R | GGTGTCCGGGTTTCTGTTCA |
|  |  |  |  |
| *lipf* | XM_015962494 | lipf-F | TCAGTGTGTGCGTTGTTCCT |
|  |  | lipf-R | GAGCCGTCAGCGTCAGAATA |
|  |  |  |  |
| ***ISH*** |  |  |  |
|  |  |  |  |
| *apoa1* | XM_015950135 | *T3*-apoa1 -S | *AATTAACCCTCACTAAAGGG* TGCATTCTGGGACTACGTTGG |
|  |  | *T7*-apoa1 -AS | *TAATACGACTCACTATAGGG* GTCAACATAGGGGGTTGCCA |
